# Anomaly formulas for the complex-valued analytic torsion on compact bordisms

**DOI:** 10.1016/j.difgeo.2013.04.003

**Published:** 2013-06

**Authors:** Osmar Maldonado Molina

**Affiliations:** Department of Mathematics, University of Vienna, Nordbergstrasse 15, A-1090 Vienna, Austria

**Keywords:** 58J52, 57R20, Analytic torsion, Bordisms, Bilinear form, Laplacians, Anomaly formulas, Heat trace asymptotic expansion

## Abstract

We extend the complex-valued analytic torsion, introduced by Burghelea and Haller on closed manifolds, to compact Riemannian bordisms. We do so by considering a flat complex vector bundle over a compact Riemannian manifold, endowed with a fiberwise nondegenerate symmetric bilinear form. The Riemmanian metric and the bilinear form are used to define non-selfadjoint Laplacians acting on vector-valued smooth forms under absolute and relative boundary conditions. In order to define the complex-valued analytic torsion in this situation, we study spectral properties of these generalized Laplacians. Then, as main results, we obtain so-called anomaly formulas for this torsion. Our reasoning takes into account that the coefficients in the heat trace asymptotic expansion associated to the boundary value problem under consideration, are locally computable. The anomaly formulas for the complex-valued Ray–Singer torsion are derived first by using the corresponding ones for the Ray–Singer metric, obtained by Brüning and Ma on manifolds with boundary, and then an argument of analytic continuation. In odd dimensions, our anomaly formulas are in accord with the corresponding results of Su, without requiring the variations of the Riemannian metric and bilinear structures to be supported in the interior of the manifold.

## 0 Introduction

In this paper, we denote by $\left(M,\partial_{+}M,\partial_{-}M\right)$ a compact Riemannian bordism. That is, *M* is a compact Riemannian manifold of dimension *m*, with Riemannian metric *g*, whose boundary ∂*M* is the disjoint union of two closed submanifolds $\partial_{+}M$ and $\partial_{-}M$. For *E* a flat complex vector bundle over *M*, we consider generalized Laplacians acting on the space Ω(M;E) of *E*-valued smooth differential forms on *M* satisfying absolute boundary conditions on $\partial_{+}M$ and relative boundary conditions on $\partial_{-}M$.

We study the *complex-valued* Ray–Singer torsion on $\left(M,\partial_{+}M,\partial_{-}M\right)$. This torsion was introduced by Burghelea and Haller on closed manifolds, see [4] and [5], as a complex-valued version for the real-valued Ray–Singer torsion, originally studied by Ray and Singer in [21] for unitary flat vector bundles on closed manifolds. Our main results are Theorem 2 and Theorem 3. In Theorem 3, we provide so-called *anomaly formulas* providing a logarithmic derivative for the complex-valued analytic torsion on compact Riemannian bordisms and its proof is based on the work by Brüning and Ma in [8] for the real-valued Ray–Singer torsion on manifolds with boundary.

The classical (*real-valued*) Ray–Singer analytic torsion, see [21], [17], [10], [19] and others, is defined in terms of a selfadjoint Laplacian $\Delta_{E,g,h}$, constructed by using a Hermitian metric on the bundle, the Riemannian metric *g* and a flat connection $\nabla^{E}$ on *E*. In this paper $\Delta_{E,g,h}$ is referred as the *Hermitian Laplacian*. In [7], Bismut and Zhang interpreted the analytic torsion as a Hermitian metric in certain determinant line, and called it the Ray–Singer metric, see also [9]. In this paper, we also adopt this approach. The Ray–Singer metric on manifolds with boundary has been intensively studied by several authors, among them [21], [10], [19], [20], [17], [11], [8], [9]. In particular, we are interested in the work of Brüning and Ma in [8], where the variation of the Ray–Singer metric, with respect to smooth variations on the underlying Riemannian and Hermitian metrics, was computed.

In order to define the *complex-valued* Ray–Singer torsion, we assume *E* admits a fiberwise nondegenerate symmetric bilinear form *b* and we proceed as in [4]. The bilinear form *b* and the Riemannian metric *g* induce a nondegenerate symmetric bilinear form on Ω(M;E) which is denoted by $\beta_{g,b}$. With this data, one constructs generalized Laplacians $\Delta_{E,g,b}:\Omega (M;E)\to \Omega (M;E)$, also referred as *bilinear Laplacians*. These generalized Laplacians are formally symmetric, with respect to $\beta_{g,b}$ on the space of smooth forms satisfying the boundary conditions specified above.

In Section 1, we use known theory on boundary value problems for differential operators to treat ellipticity, regularity and spectral properties for $\Delta_{E,g,b}$. In particular, under the specified elliptic boundary conditions, $\Delta_{E,g,b}$ extends to a not necessarily selfadjoint closed unbounded operator in the $L^{2}$-norm, it has compact resolvent and discrete spectrum, all its eigenvalues are of finite multiplicity, its (generalized) eigenspaces contain smooth differential forms only and the restriction of $\beta_{g,b}$ to each of these is also a nondegenerate bilinear form. Proposition 2 gives Hodge decomposition results in this setting, which are analog to the Hermitian situation, described for instance in [10], [19], [17] and more recently in [9]. Section 1 ends with Proposition 3 stating that the 0-generalized eigenspace of $\Delta_{E,g,b}$ *still* computes relative cohomology $H(M,\partial_{-}M;E)$, without necessarily being isomorphic to it.

In Section 2, we recall generalities on the coefficients of the heat kernel asymptotic expansion for an elliptic boundary value problem. These coefficients are spectral invariants and locally computable as polynomial functions in the jets of the symbols of the operators under consideration, see [14], [22], [23], [24]. This fact provides the key ingredient in the proofs of Theorem 2, leading to Theorem 3. In [8], based on the computation of the coefficients of the constant terms in the heat trace asymptotic expansion for the Hermitian Laplacian under absolute boundary conditions, Brüning and Ma obtained anomaly formulas for the Ray–Singer metric. First, we use Poincaré duality in terms of Lemma 6, to infer from [8], the corresponding coefficients for the Hermitian Laplacian under relative boundary conditions and then we derive those corresponding to Hermitian Laplacian on the bordism $\left(M,\partial_{+}M,\partial_{-}M\right)$ under absolute and relative boundary conditions, see Proposition 5 and Theorem 1. We point out here that the anomaly formulas for the Ray–Singer metric in Theorem 1 were also obtained by Brüning and Ma in [9] continuing their work in [8]. Next, in Lemma 10, we point out the holomorphic dependance of these coefficients on a complex parameter. Finally, an analytic continuation argument allows one to deduce the infinitesimal variation of these quantities for the bilinear Laplacian on the bordism $\left(M,\partial_{+}M,\partial_{-}M\right)$ from those corresponding to the Hermitian one, see Theorem 2.

In Section 3, we use the results from Section 1 and Section 2 to define the complex-valued analytic torsion on a compact Riemannian bordism. Following the approach in [4], we obtain a nondegenerate bilinear form on the determinant line $\det(H(M,\partial_{-}M;E))$, denoted by $\tau_{E,g,b}(0)$ and induced by the restriction of $\beta_{g,b}$ to the generalized 0-eigenspace of $\Delta_{E,g,b}$. The (inverse square of) the complex-valued Ray–Singer torsion for manifolds with boundary is

$$\tau_{E,g,b}^{\mathrm{RS}}:=\tau_{E,g,b}(0)\cdot \prod_{p}{\left(\det^{\prime }(\Delta_{E,g,b,p})\right)}^{{\left(-1\right)}^{p}p},$$

where the product above is, in this situation, a non-zero complex number with $\det^{\prime }(\Delta_{E,g,b,p})$ being the *ζ*-regularized product of all non-zero eigenvalues of $\Delta_{E,g,b,p}$. For closed manifolds, the variation of the complex analytic Ray–Singer torsion, with respect to smooth changes on the metric *g* and the bilinear form *b*, has been obtained in [4, Sections 7 and 8]. Burghelea and Haller obtained in [4, Theorem 4.2] a geometric invariant by introducing appropriate correction terms. In [25], by using techniques from [26], [27], [10], [19], Su generalized the complex-valued analytic Ray–Singer torsion to the situation in which $\partial_{+}M\neq \emptyset$ (or $\partial_{-}M\neq \emptyset$). Also in [25], Su proved that in odd dimensions, the complex-valued analytic torsion does depend neither on smooth variations of the Riemannian metric nor on smooth variations of the bilinear form, as long as these are compactly supported in the interior of *M*. This section ends with Theorem 3, which gives formulas for the variation of the complex-valued analytic Ray–Singer torsion with respect to smooth variations of the metric and the bilinear form. In analogy with the results in [4], the anomaly formulas for the complex-valued Ray–Singer torsion are obtained by using the results for the coefficients of the constant term in the heat trace asymptotic expansion for the bilinear Laplacian obtained in Section 2.

In Appendix A, for the readerʼs convenience, we recall some formalism leading to the characteristic forms appearing in the anomaly formulas stated in Proposition 4, Proposition 5, Theorem 1, Theorem 2 and Theorem 3.

The anomaly formulas given in Theorem 3 generalize the ones obtained by Burghelea and Haller in the closed situation in [4], and also the ones in [25] by Su in odd dimensions: they do not longer require *g* and *b* to be constant in a neighborhood of the boundary and both kind of boundary conditions are considered at the same time.

## 1 Bilinear Laplacians and Hodge decomposition on bordisms

### 1.1 Some background and notation

Let $\left(M,\partial_{+}M,\partial_{-}M\right)$ be a compact Riemannian bordism of dimension *m*. More precisely, *M* is a compact connected not necessarily orientable smooth manifold of dimension *m* with Riemannian metric *g*, whose boundary ∂*M* is the disjoint union of two closed submanifolds, $\partial_{+}M$ and $\partial_{-}M$, and it inherits the Riemannian metric from *M*. We do not require the metric to satisfy any condition near the boundary. We denote by *TM* and $T^{⁎}M$ (resp. T∂M and $T^{⁎}\partial M$) the tangent and cotangent bundle of *M* (resp. ∂*M*) respectively. We denote by $\varsigma_{\mathrm{in}}$ the *geodesic unit inwards pointing normal vector field* on the boundary. Let $\Theta_{M}$ (resp. $\Theta_{\partial M}$) be the orientation bundle of *TM* (resp. T∂M), considered as the flat real line bundle $\det(T^{⁎}M)\to M$ (resp. $\det(T^{⁎}\partial M)\to \partial M$) with transition functions {±1}, endowed with the unique flat connection specified by the de-Rham differential on (twisted) forms, see [3, p. 88]. For the canonical embedding i:∂M↪M, we write $\Theta_{M}{|}_{\partial M}:=i^{⁎}\Theta_{M}$ and, as real line bundles over ∂*M*, $\Theta_{M}{|}_{\partial M}$ and $\Theta_{\partial M}$ are identified as follows: over the boundary, a section *β* of $\det(T^{⁎}\partial M)$ is identified with the section $-\varsigma^{\mathrm{in}}\wedge \beta$ of $\det(T^{⁎}M){|}_{\partial M}$, where $\varsigma^{\mathrm{in}}:=g(\cdot ,\varsigma_{\mathrm{in}})$ is the 1-form dual to $\varsigma_{\mathrm{in}}$. For *TM* and T∂M, the corresponding Levi-Cività connections are denoted by ∇ and by $\nabla^{\partial }$ respectively. Recall the Hodge ⋆-operator ${⋆}_{q}:={⋆}_{g,q}:\Omega^{q}(M)\to \Omega^{m-q}(M;\Theta_{M})$, i.e., the linear isomorphism defined by $\alpha \wedge ⋆\alpha^{\prime }={\langle \alpha ,\alpha^{\prime }\rangle }_{g}{\mathrm{vol}}_{g}(M)$, for $\alpha ,\alpha^{\prime }\in \Omega^{q}(M)$ and 0≤q≤m, where ${\mathrm{vol}}_{g}(M)\in \Omega^{m}(M;\Theta_{M})$ is the volume form of *M*.

In this paper, we consider a flat complex vector bundle *E* over *M*, with a flat connection $\nabla^{E}$, and denote by Ω(M;E) be the space of *E*-valued smooth differential forms on *M*, endowed with the de-Rham differential $d_{E}:=d_{\nabla^{E}}$. Moreover, assume *E* is endowed with a fiberwise nondegenerate symmetric bilinear form *b*. We denote by $E^{\prime }$ the flat complex vector bundle dual to *E* with the induced flat connection $\nabla^{E^{\prime }}$ and bilinear form $b^{\prime }$ dual to $\nabla^{E}$ and *b* respectively. Recall that one is always able to fix a (positive definite) Hermitian structure on *E* (in Section 2.3, we choose for instance a Hermitian structure compatible with the nondegenerate symmetric bilinear form). By choosing a Hermitian structure on *E* and using the Riemannian metric on *M*, consider the induced $L^{2}$-norm on Ω(M;E) and denote by $L^{2}(M;E)$ its $L^{2}$-completion. Recall that $L^{2}(M;E)$ is independent the chosen Hermitian and Riemannian structures.

### 1.2 Generalized Laplacians on compact bordisms

As a first step to define the complex-valued analytic torsion on a compact bordism, we recall certain generalized Laplacians which were introduced in [4] on closed manifolds. The nondegenerate symmetric bilinear form *b* on *E* and the Riemannian metric *g* on *M* permit to define a nondegenerate symmetric bilinear form on Ω(M;E) by

$$\beta_{g,b}(v,w):=\int_{M}\mathrm{Tr}(v\wedge {⋆}_{b}w)$$

where $\mathrm{Tr}:\Omega (M,E⊗E^{\prime }⊗\Theta_{M})\to \Omega (M;\Theta_{M})$ is the trace map, induced by the canonical pairing between the bundles *E* and $E^{\prime }$, and the map

$${⋆}_{b,q}:={⋆}_{q}⊗b:\Omega^{q}(M;E)\to \Omega^{m-q}\left(M;E^{\prime }⊗\Theta_{M}\right)$$

is defined by using the Hodge ⋆-operator ${⋆}_{q}$ and the isomorphism of vector bundles between *E* and $E^{\prime }$, specified by the bilinear form *b*, also denoted by the same symbol. Thus, one defines $d_{E,g,b,q}^{♯}:\Omega^{q}(M;E)\to \Omega^{q-1}(M;E)$ by

(1) $$d_{E,g,b,q}^{♯}:={\left(-1\right)}^{q}{{⋆}_{b,q-1}}^{-1}\;d_{E^{\prime }⊗\Theta_{M},m-q}{⋆}_{b,q},$$

where ${{⋆}_{b,q-1}}^{-1}$ is the inverse of ${⋆}_{b,q-1}$ and $d_{E^{\prime }⊗\Theta_{M}}$ is the de-Rham differential on $\Omega (M;E^{\prime }⊗\Theta_{M})$ induced by the dual connection on $E^{\prime }$. It can easily be checked that $d_{E,g,b}^{♯}$ is a codifferential on Ω(M;E). In this way, the operator

(2) $$\Delta_{E,g,b,q}:=d_{E,q-1}\;d_{E,g,b,q}^{♯}+d_{E,g,b,q+1}^{♯}\;d_{E,q}:\Omega^{q}(M;E)\to \Omega^{q}(M;E),$$

is an operator of Laplace type, or generalized Laplacian in the sense that its principal symbol is a scalar positive real number, i.e., $\Delta_{E,g,b}$ is elliptic. For simplicity, the operator $\Delta_{E,g,b}$ in (2) will be called the *bilinear Laplacian*. A straightforward use of Stokesʼ Theorem leads to the Greenʼs formulas:

(3) $$\beta_{g,b}(d_{E}\;v,w)-\beta_{g,b}\left(v,d_{E,g,b}^{♯}w\right)=\int_{\partial M}i^{⁎}\left(\mathrm{Tr}(v\wedge {⋆}_{b}w)\right),$$

$$\beta_{g,b}(\Delta_{E}v,w)-\beta_{g,b}(v,\Delta_{E}w)=\int_{\partial M}i^{⁎}\left(\mathrm{Tr}\left(d_{E,g,b}^{♯}v\wedge {⋆}_{b}w\right)\right)-\int_{\partial M}i^{⁎}\left(\mathrm{Tr}(w\wedge {⋆}_{b}\;d_{E}\;v)\right)-\int_{\partial M}i^{⁎}\left(\mathrm{Tr}\left(d_{E,g,b}^{♯}w\wedge {⋆}_{b}v\right)\right)+\int_{\partial M}i^{⁎}\left(\mathrm{Tr}(v\wedge {⋆}_{b}\;d_{E}\;w)\right),$$

for v,w∈Ω(M;E).

### 1.3 Boundary conditions

In order to study analytic and spectral properties of $\Delta_{E,g,b}$, we impose elliptic boundary conditions. We denote by $i_{\pm }:\partial_{\pm }M↪M$ the canonical embedding of $\partial_{\pm }M$ into *M* respectively. For a form w∈Ω(M;E), we say that *w* satisfies *relative boundary conditions* on $\partial_{-}M$ if $i_{-}^{⁎}w=0$ and $i_{-}^{⁎}d_{E,g,b}^{♯}\;w=0$ and *w* satisfies *absolute boundary conditions* on $\partial_{+}M$ if $i_{+}^{⁎}{⋆}_{b}w=0$ and $i_{+}^{⁎}\;d_{E^{\prime }⊗\Theta_{M},g,b}^{♯}{⋆}_{b}w=0$. The space of smooth forms satisfying relative boundary conditions on $\partial_{-}M$ *and* absolute boundary conditions on $\partial_{+}M$ is

(4) $$\Omega (M;E){|}_{B}:=\left\{w\in \Omega (M;E)|\begin{array}{cc} i_{+}^{⁎}{⋆}_{b}w=0, & i_{-}^{⁎}w=0\\ i_{+}^{⁎}\;d_{E^{\prime }⊗\Theta_{M},g,b}^{♯}{⋆}_{b}w=0, & i_{-}^{⁎}d_{E,g,b}^{♯}w=0 \end{array}\right\}.$$

For simplicity, a form satisfying boundary conditions in (4) will be referred as satisfying *absolute/relative boundary conditions on* $\left(M,\partial_{+}M,\partial_{-}M\right)$. The integrants on the right of formulas in (3) vanish, on forms in $\Omega (M;E){|}_{B}$. The boundary conditions in (4) are an example of *mixed boundary conditions*, which provide elliptic boundary conditions for operators of Laplace type, see [13].

Now we describe boundary operators implementing the boundary conditions in (4). Consider $E_{\pm }:=i_{\pm }^{⁎}E$ and for 1≤q≤m define

(5) $$\begin{array}{cccc} B_{E,g,b}: & \Omega^{q}(M;E) & \to & \Omega^{q-1}(\partial_{+}M;E_{+})⊕\Omega^{q}(\partial_{+}M;E_{+})\\ & & & ⊕\Omega^{q}(\partial_{-}M;E_{-})⊕\Omega^{q-1}(\partial_{-}M;E_{-})\\ & w & \mapsto & (B_{+}w,B_{-}w), \end{array}$$

where the operators

(6) $$\begin{array}{cccc} B_{-}: & \Omega^{q}(M;E) & \to & \Omega^{q}(\partial_{-}M;E_{-})⊕\Omega^{q-1}(\partial_{-}M;E_{-})\\ & w & \mapsto & \left(B_{-}^{0}w,B_{-}^{1}w\right), \end{array}$$

$$\begin{array}{cccc} B_{+}: & \Omega^{q}(M;E) & \to & \Omega^{q-1}(\partial_{+}M;E_{+})⊕\Omega^{q}(\partial_{+}M;E_{+})\\ & w & \mapsto & \left(B_{+}^{0}w,B_{+}^{1}w\right) \end{array}$$

are respectively defined in terms of

(7) $$B_{-}^{0}w:=i_{-}^{⁎}w,\;B_{-}^{1}w:=i_{-}^{⁎}d_{E,g,b}^{♯}w,$$

$$B_{+}^{0}w:={{⋆}_{b}^{\partial M}}^{-1}\left(i_{+}^{⁎}{⋆}_{b}w\right),\;B_{+}^{1}w:={{⋆}_{b}^{\partial M}}^{-1}\left(i_{+}^{⁎}\;d_{E^{\prime }⊗\Theta_{M},g,b^{\prime }}^{♯}{⋆}_{b}w\right).$$

A form *w* satisfies the boundary conditions, i.e., $w\in \Omega (M;E){|}_{B}$, if and only if Bw=0.

Lemma 1 *For a subspace*X⊆Ω(M;E)*, denote by*$X{|}_{B}:=\{w\in X|Bw=0\}$*the space of smooth forms in*X*which satisfy the boundary conditions specified by the vanishing of the operator*$B\in \{B_{\pm }^{0},B_{\pm }^{1},B_{\pm },B\}$*. Set*

(8) $$X{|}_{B^{0}}:=X{|}_{B_{-}^{0}}\cap X{|}_{B_{+}^{0}}.$$

*Then the following assertions hold*:

(a) $X{|}_{B}=X{|}_{B^{0}}\cap X{|}_{B_{-}^{1}}\cap X{|}_{B_{+}^{1}}$*and*$X{|}_{B}\subset X{|}_{B^{0}}\subset X{|}_{B_{-}^{0}}$*,*
(b) $d_{E}(\Omega (M;E){|}_{B_{-}^{0}})\subset \Omega (M;E){|}_{B_{-}^{0}}$*,*
(c) $d_{E}(\Omega (M;E){|}_{B})\subset \Omega (M;E){|}_{B^{0}}$*and*$d_{E,g,b}^{♯}(\Omega (M;E){|}_{B})\subset \Omega (M;E){|}_{B^{0}}$*,*
(d) *if*$v\in \Omega (M;E){|}_{B_{-}^{0}}$*and*$w\in \Omega (M;E){|}_{B}$*then*$\beta_{g,b}(d_{E}\;v,d_{E,g,b}^{♯}\;w)=0$*,*
(e) *if*$v,w\in \Omega (M;E){|}_{B^{0}}$*, then*$\beta_{g,b}(d_{E}\;v,w)=\beta_{g,b}(v,d_{E,g,b}^{♯}\;w)$*,*
(f) *if*$v,w\in \Omega (M;E){|}_{B}$*, then*$\beta_{g,b}(\Delta_{E,g,b}v,w)=\beta_{g,b}(v,\Delta_{E,g,b}w)$*.*

Proof The first assertion is obvious. The remaining assertions follow from (8), (4), the Greenʼs formulas in (3) and straightforward manipulations coming from the definition of the operators and spaces above.  □

### 1.4 Boundary conditions and Poincaré duality

Consider the Riemannian bordism $\left(M,\partial_{+}M,\partial_{-}M\right)$. The boundary value problem specified by the operator $\Delta_{E,g,b}$ acting on the space $\Omega (M;E){|}_{B}$ as defined by (4), will be denoted by

(9) $${\left[\Delta ,B\right]}_{\left(M,\partial_{+}M,\partial_{-}M\right)}^{E,g,b}.$$

Let us denote by ${\left(M,\partial_{+}M,\partial_{-}M\right)}^{\prime }:=(M,\partial_{-}M,\partial_{+}M)$ the *dual bordism* to $\left(M,\partial_{+}M,\partial_{-}M\right)$. Then, we are interested in ${\left[\Delta ,B\right]}_{{\left(M,\partial_{+}M,\partial_{-}M\right)}^{\prime }}^{E^{\prime }⊗\Theta ,g,b^{\prime }}$ the dual boundary value problem to (9), corresponding to the bilinear Laplacian $\Delta_{E^{\prime },g,b^{\prime }}$ acting on $E^{\prime }⊗\Theta_{M}$-valued forms (where the flat complex vector bundle $E^{\prime }$ is endowed with the dual connection $\nabla^{E^{\prime }}$ and dual bilinear form $b^{\prime }$) under the boundary conditions specified by the vanishing of the boundary operator $B^{\prime }$, i.e., the same operator from (5) but associated to ${\left(M,\partial_{+}M,\partial_{-}M\right)}^{\prime }$. The boundary value problem in (9) is naturally intertwined with its dual one by means of the Hodge ⋆-operator. Indeed, by the very definition of these operators, we have the equality

$${⋆}_{b}d_{E,g,b}^{♯}d_{E}=d_{E^{\prime }⊗\Theta_{M}}\;d_{E^{\prime }⊗\Theta_{M},g,b^{\prime }}^{♯}{⋆}_{b}$$

so that

$${⋆}_{b}\Delta_{E,g,b}=\Delta_{E^{\prime }⊗\Theta_{M},g,b^{\prime }}{⋆}_{b},$$

and

$$w\in \Omega^{q}(M;E){|}_{B}\;⟺\;{⋆}_{b}w\in {\Omega^{m-q}\left(M;E^{\prime }⊗\Theta_{M}\right)|}_{B^{\prime }}.$$

That is, the Hodge-${⋆}_{b}$-operator intertwines the roles of $\partial_{+}M$ and $\partial_{-}M$ in (9) and its dual.

As a special case, if $\partial_{+}M=\partial M$ and $\partial_{-}M=\emptyset$ (resp. $\partial_{+}M=\emptyset$ and $\partial_{-}M=\partial M$), then ${\left[\Delta ,B\right]}_{\left(M,\partial M,\emptyset \right)}^{E,g,b}$, (resp. ${\left[\Delta ,B\right]}_{\left(M,\emptyset ,\partial M\right)}^{E,g,b}$) is the boundary value problem where absolute (resp. relative) boundary conditions *only* are imposed on ∂*M*.

### 1.5 Hermitian boundary value problems

We recall some facts for the Hermitian situation. By using a Hermitian structure *h* on *E*, instead of the bilinear form *b*, all over in the considerations above, one has ${《v,w》}_{g,h}:=\int_{M}\mathrm{Tr}(v\wedge {⋆}_{h}w)$ a Hermitian product on Ω(M;E), where ${⋆}_{h}$ is in this case a fiber-wise complex anti-linear isomorphism induced by *h* and ${⋆}_{g}$. Then, associated to this data, one considers a differential $d_{E}$, a codifferential $d_{E,g,h}^{⁎}$ and a Laplacian

$$\Delta_{E,g,h}:=d_{E}\;d_{E,g,h}^{⁎}+d_{E,g,h}^{⁎}d_{E}:\Omega (M;E)\to \Omega (M;E),$$

which is formally selfadjoint with respect to ${《v,w》}_{g,h}$, under absolute/relative boundary conditions on $\left(M,\partial_{+}M,\partial_{-}M\right)$. Let $\Omega (M;E){|}_{B}^{h}$ be the space of *E*-valued smooth forms satisfying absolute/relative boundary conditions on $\left(M,\partial_{+}M,\partial_{-}M\right)$ defined as in (4) but using instead the Hermitian form *h*. In order to distinguish this problem from the bilinear one, we refer to it as the *Hermitian boundary value problem*.

The Hermitian boundary value problem is an elliptic boundary value problem, see [12] and [13]. This permits one to consider $\Delta_{E,g,h}$, as an unbounded operator in the $L^{2}$-norm and extend it to a selfadjoint operator with domain of definition being the $H_{2}$-Sobolev closure of $\Omega (M;E){|}_{B}^{h}$; see [17], [10], [19], [12], [13]. In particular, in this Hermitian setting, there are well-known Hodge decomposition results. For instance, if $H_{\Delta_{B}}^{q}(M;E)$ is the space $\ker(\Delta_{E,g,h})\cap \Omega^{q}(M;E){|}_{B}^{h}$ of *q*-*Harmonic forms* satisfying boundary conditions, then [17, Theorem 1.10] (see also [19, p. 239]) states that for each $v\in \Omega^{q}(M;E){|}_{B^{0}}^{h}$, there exist unique $v_{0}\in H_{\Delta_{B}}^{q}(M;E)$, $v_{1}\in d_{E}(\Omega^{q-1}(M;E){|}_{B^{0}}^{h})$ and $v_{2}\in d_{E,g,h}^{⁎}(\Omega^{q+1}(M;E){|}_{B^{0}}^{h})$ such that $v=v_{0}+v_{1}+v_{2}$, where we have used the notation suggested in (8) associated to *h*. Moreover, the Hodge–de-Rham tells us that relative cohomology exactly coincides with the space of Harmonic forms of the Hermitian Laplacian:

(10) $$H_{\Delta_{B}}^{q}(M;E)≅H^{q}(M,\partial_{-}M;E).$$

In the bilinear setting, the isomorphism in (10) does no longer holds, but we have instead Proposition 3 below. One uses the isomorphism in (10) to define the Ray–Singer metric on manifolds with boundary, as a Hermitian metric on the determinant line in (relative) cohomology. This problem has been studied by many authors, see for instance [21], [17], [10], [19], [11], [8], [9]. In particular, we are interested in the work by Brüning and Ma in [8], where the case $\partial_{-}M=\emptyset$ was studied.

### 1.6 The spectrum of the bilinear Laplacian

Consider the boundary valued problem ${\left[\Delta ,B\right]}_{\left(M,\partial_{+}M,\partial_{-}M\right)}^{E,g,b}$. Here we denote by $H_{s}(M;E)$ for s≥0, the corresponding Sobolev completions of Ω(M;E) with respect to a Hermitian metric on *E*. By [16, Section 20.1] and [1, Chapter 1], the operators $\Delta_{E,g,b}$ and $B_{E,g,b}$ extend as a linear *bounded* operators

(11) $$\Delta_{E,g,b}:H_{2}(M;E)\to L^{2}(M;E)$$

and

(12) $$B_{E,g,b}:H_{2}(M,E)\to H_{\frac{1}{2}}(\partial M;E{|}_{\partial M})⊕H_{\frac{3}{2}}(\partial M,E{|}_{\partial M})$$

respectively and again these are independent on the chosen Hermitian structure.

By the $L^{2}$*-realization* of the bilinear Laplacian is understood the same operator in (11) but considered as the *unbounded* operator in $L^{2}(M;E)$

(13) $$\Delta_{B}:D(\Delta_{B})\subset L^{2}(M;E)\to L^{2}(M;E)$$

with domain of definition

(14) $$D(\Delta_{B}):={\bar{\Omega (M;E){|}_{B}}}^{H_{2}}.$$

The boundary value problem ${\left[\Delta ,B\right]}_{\left(M,\partial_{+}M,\partial_{-}M\right)}^{E,g,b}$ is elliptic with respect to the cone C\(0,∞), see [13, Lemma 1.5.3]. Boundary ellipticity guarantees the existence of elliptic estimates, see [1, Theorem 6.3.1] and [16, Theorem 20.1.2]. Then, elliptic estimates permit one to conclude that the $L^{2}$-realization of the bilinear Laplacian is a *closed* unbounded operator in $L^{2}(M;E)$, which coincides with the $L^{2}$-closure extension of

$$\Delta_{E,g,b}:\Omega (M;E){|}_{B}\subset L^{2}(M;E)\to \Omega (M;E)\subset L^{2}(M;E),$$

regarded as unbounded operator in $L^{2}(M;E)$.

Lemma 2 *Let*$\Delta_{B}$*be the unbounded operator with domain of definition*$D(\Delta_{B})$*given in*(14)*. This operator is densely defined in*$L^{2}(M;E)$*, possesses a non-empty resolvent set, its resolvent is compact and its spectrum is discrete. More precisely, for every*θ>0*, there exists*R>0*such that*$B_{R}(0)$*, the closed ball in*C*centered at 0 and radius R, contains at most a finite subset of*$\mathrm{Spec}(\Delta_{B})$*and the remaining part of the spectrum is entirely contained in the sector*

$$\Lambda_{R,\theta }:=\left\{z\in C|-\theta <\arg(z)<\theta \text{and}|z|\geq R\right\}.$$

*Furthermore, for every*$\lambda \notin \Lambda_{R,\theta }$*large enough, there is*C>0*, for which*$\left\|{\left(\Delta_{B}-\lambda \right)}^{-1}{\|}_{L^{2}}\leq C/|\lambda \right|$*.*

Proof This follows from boundary ellipticity with respect to the conical set C\(0,∞). For a detailed discussion on this result (which holds also in the more general setting of pseudo-differential boundary value problems for operators), we refer the reader to [15, Theorem 3.3.2, Corollary 3.3.3 and Remark 3.3.4] (see also [15, Section 1.5]).  □

### 1.7 Generalized eigenspaces

By Lemma 2, $\mathrm{Spec}(\Delta_{B})$ is discrete and then, for each $\lambda \in \mathrm{Spec}(\Delta_{B})$, we choose γ(λ) a closed counter-clock-wise oriented curve surrounding *λ* as the unique point of $\mathrm{Spec}(\Delta_{B})$. Consider the corresponding Riesz or spectral projection:

(15) $$\begin{array}{cccc} P_{\Delta_{B}}(\lambda ): & L^{2}(M;E) & \to & D(\Delta_{B})\subset L^{2}(M;E)\\ & w & \mapsto & -{\left(2\pi i\right)}^{-1}\int_{\gamma (\lambda )}{\left(\Delta_{B}-\mu \right)}^{-1}w\;d\mu . \end{array}$$

The integral above in (15) converges uniformly in the $L^{2}$-norm as the limit of Riemann sums, since the function $x\mapsto {\left(\Delta_{B}-x\right)}^{-1}$ is analytic in a neighborhood of γ(λ). The image of $P_{\Delta_{B}}(\lambda )$ in $L^{2}(M;E)$ is denoted by

$$\Omega_{\Delta_{B}}(M;E)(\lambda ):=P_{\Delta_{B}}(\lambda )\left(L^{2}(M;E)\right).$$

Since the resolvent of $\Delta_{B}$ is compact, the operator $P_{\Delta_{B}}(\lambda )$ is bounded on $L^{2}(M;E)$, and $\Omega_{\Delta_{B}}(M;E)(\lambda )$ is of finite dimension, see [18, Theorem 6.29]. The image of the complementary projection to $P_{\Delta_{B}}(\lambda )$ on $L^{2}(M;E)$ is denoted by

Im(Id−PΔB(λ)):=(Id−PΔB(λ))(L2(M;E)).

Then the space $L^{2}(M;E)$ decomposes as a direct sum of Hilbert spaces compatible with the projections $P_{\Delta_{B}}(\lambda )$ and $\left(\mathrm{Id}-P_{\Delta_{B}}(\lambda )\right)$. More precisely, the following lemma is a direct application of [18, Theorem 6.17].

Lemma 3 *Consider the unbounded operator*$\left(\Delta_{B},D(\Delta_{B})\right)$*from*(13)*. For*$\lambda \in \mathrm{Spec}(\Delta_{B})$*consider the corresponding spectral projection*$P_{\Delta_{B}}(\lambda )$*. Then*$\Delta_{B}$*commutes with*$P_{\Delta_{B}}(\lambda )$; *that is, for*$u\in D(\Delta_{B})$*, we have*

$$P_{\Delta_{B}}(\lambda )u\in D(\Delta_{B})\;\text{and}\;P_{\Delta_{B}}(\lambda )\Delta_{B}u=\Delta_{B}P_{\Delta_{B}}(\lambda )u.$$

*The space*$L^{2}(M;E)$*decomposes as*

L2(M;E)≅ΩΔB(M;E)(λ)⊕Im(Id−PΔB(λ)),

*such that*

$$P_{\Delta_{B}}(\lambda )\left(D(\Delta_{B})\right)\subset D(\Delta_{B}),$$

$$\Delta_{B}\left(\Omega_{\Delta_{B}}(M;E)(\lambda )\right)\subset \Omega_{\Delta_{B}}(M;E)(\lambda ),$$

ΔB(Im(Id−PΔB(λ))∩D(ΔB))⊂Im(Id−PΔB(λ)).

*The operator*

(16) $$\Delta_{B}{|}_{\Omega_{\Delta_{B}}(M;E)(\lambda )}:\Omega_{\Delta_{B}}(M;E)(\lambda )\to \Omega_{\Delta_{B}}(M;E)(\lambda ),$$

*is bounded on*$\Omega_{\Delta_{B}}(M;E)(\lambda )$*,*$\mathrm{Spec}(\Delta_{B}{|}_{\Omega_{\Delta_{B}}(M;E)(\lambda )})=\{\lambda \}$*and the operator*

(17) (ΔB−λ)|D((ΔB−λ)|Im(Id−PΔB(λ))):::D((ΔB−λ)|Im(Id−PΔB(λ)))→Im(Id−PΔB(λ)),

*with domain of definition*

D((ΔB−λ)|Im(Id−PΔB(λ)))::=:Im(Id−PΔB(λ))∩D(ΔB)⊂L2(M;E),

*is invertible, i.e., the spectrum of*ΔB|Im(Id−PΔB(λ))*is exactly*$\mathrm{Spec}(\Delta_{B})\backslash \{\lambda \}$*.*

The operator $\Delta_{B}{|}_{\Omega_{\Delta_{B}}(M;E)(\lambda )}$ in (16) being bounded, its spectrum containing *λ* only and $\Omega_{\Delta_{B}}(M;E)(\lambda )$ being of finite dimension, the operator $(\Delta_{B}-\lambda ){|}_{\Omega_{\Delta_{B}}(M;E)(\lambda )}$ is nilpotent.

Commutativity of $P_{\Delta_{B}}(\lambda )$ with $\Delta_{B}$ on its domain $D(\Delta_{B})$, invariance of $\Omega_{\Delta_{B}}(M;E)(\lambda )$ under $\Delta_{B}$, and (iteratively) using elliptic estimates with Sobolev embedding, one has $\Omega_{\Delta_{B}}(M;E)(\lambda )\subset \Omega (M;E){|}_{B}\subset \Omega (M;E)$. Thus each *λ*-eigenspace can be described as

$$\Omega_{\Delta_{B}}(M;E)(\lambda )=\left\{w\in \Omega (M;E){|}_{B}|\begin{array}{cc} {\left(\Delta_{E,g,b}-\lambda \right)}^{n}w\in \Omega (M;E){|}_{B}, & \forall n\geq 0,\\ \exists N\in N\text{ s.t. }{\left(\Delta_{E,g,b}-\lambda \right)}^{n}w=0, & \forall n\geq N \end{array}\right\}.$$

Lemma 4 *The space*$\Omega_{\Delta_{B}}(M;E)(\lambda )$*is invariant under*$d_{E}$*and*$d_{E,g,b}^{♯}$*.*

Proof We show that $\Omega_{\Delta_{B}}(M;E)(\lambda )$ is invariant under $d_{E}$ and $d_{E,g,b}^{♯}$. Since $\Omega_{\Delta_{B}}(M;E)(\lambda )$ contains smooth differential forms only, it suffices to show that $d_{E}\;w$ satisfies the boundary condition, whenever $w\in \Omega_{\Delta_{B}}(M;E)(\lambda )$. On $\partial_{+}M$, the absolute part of the boundary, this immediately follows from ${d_{E}}^{2}=0$. Let us turn to $\partial_{-}M$, the relative part of the boundary. But, we know that the Riesz projections are well defined as bounded operators and they commute with the Laplacian on its domain of definition. That is, $\Delta_{E,g,b}w$ lies in $\Omega_{\Delta_{B}}(M;E)(\lambda )$ as well; in particular, it satisfies relative boundary conditions on $\partial_{-}M$, so that $i_{-}^{⁎}(\Delta_{E,g,b}w)=0$. Together with $i_{-}^{⁎}d_{E,g,b}^{♯}\;w=0$, this implies $i_{-}^{⁎}d_{E,g,b}^{♯}\;d_{E}\;w=0$, hence $d_{E}\;w$ also satisfies relative boundary conditions. Finally, the corresponding statement for $d_{E,g,b}^{♯}$ follows by the duality between the absolute and relative boundary operators.  □

### 1.8 Orthogonality and Hodge decomposition for smooth forms

We are interested in the space of smooth forms being in the complement image of $P_{B}(\lambda )$, which is denoted by

(18) ΩΔB(M;E)(λ)c:=Ω(M;E)∩Im(Id−PΔB(λ)).

Invertibility of the operator given in (17) and the existence of elliptic estimates imply that the restriction of $\left(\Delta_{B}-\lambda \right)$ to the space $\Omega_{\Delta_{B}}(M;E){\left(\lambda \right)}^{c}$ given in (18), satisfying boundary conditions provides, with the notation in display (8), the isomorphism

(19) $$(\Delta_{B}-\lambda ){|}_{\Omega_{\Delta_{B}}(M;E){\left(\lambda \right)}^{c}{|}_{B}}:\Omega_{\Delta_{B}}(M;E){\left(\lambda \right)}^{c}{|}_{B}\to \Omega_{\Delta_{B}}(M;E){\left(\lambda \right)}^{c}.$$

Lemma 5 *For*$\lambda \in \mathrm{Spec}(\Delta_{B})$*and*$v,w\in L^{2}(M;E)$*, we have the formula*$\beta_{g,b}(P_{\Delta_{B}}(\lambda )v,w)=\beta_{g,b}(v,P_{\Delta_{B}}(\lambda )w)$*.*

Proof Since $\beta_{g,b}$ continuously extends to a nondegenerate bilinear form on $L^{2}(M;E)$, it is enough to prove the statement on smooth forms. For v,w∈Ω(M;E) and the definition of the spectral projection in (15), we have

$$-2\pi i\beta_{g,b}\left(P_{\Delta_{B}}(\lambda )v,w\right)=\beta_{g,b}\left(\int_{\gamma_{\lambda }}{\left(\Delta_{B}-\mu \right)}^{-1}v\;d\mu ,w\right)=\int_{\gamma_{\lambda }}\beta_{g,b}\left({\left(\Delta_{B}-\mu \right)}^{-1}v,w\right)\;d\mu ,$$

where the last equality above holds, since $\int_{\gamma_{\lambda }}$ converges uniformly in the $L^{2}$-norm. Since $\gamma_{\lambda }\cap \mathrm{Spec}(\Delta_{B})=\emptyset$, we have ${\left(\Delta_{B}-\mu \right)}^{-1}w\in D(\Delta_{B})$ so that $w=(\Delta_{B}-\mu ){\left(\Delta_{B}-\mu \right)}^{-1}w$ for each $\mu \in \gamma_{\lambda }$. Now, from the isomorphism in (19), both ${\left(\Delta_{B}-\mu \right)}^{-1}v$ and ${\left(\Delta_{B}-\mu \right)}^{-1}w$ belong in fact to $\Omega_{\Delta_{B}}(M;E){\left(\lambda \right)}^{c}{|}_{B}$, so we can apply Lemma 1 and obtain

$$\beta_{g,b}\left({\left(\Delta_{B}-\mu \right)}^{-1}v,w\right)=\beta_{g,b}\left({\left(\Delta_{B}-\mu \right)}^{-1}v,(\Delta_{E,g,b}-\mu ){\left(\Delta_{B}-\mu \right)}^{-1}w\right)=\beta_{g,b}\left((\Delta_{E,g,b}-\mu ){\left(\Delta_{B}-\mu \right)}^{-1}v,{\left(\Delta_{B}-\mu \right)}^{-1}w\right)=\beta_{g,b}\left(v,{\left(\Delta_{B}-\mu \right)}^{-1}w\right);$$

that is, $\beta_{g,b}(P_{\Delta_{B}}(\lambda )v,w)=-{\left(-2\pi i\right)}^{-1}\int_{\gamma_{\lambda }}\beta_{g,b}(v,{\left(\Delta_{B}-\mu \right)}^{-1}w)\;d\mu$ and hence the equality $\beta_{g,b}(P_{\Delta_{B}}(\lambda )v,w)=\beta_{g,b}(v,P_{\Delta_{B}}(\lambda )w)$ holds.  □

Proposition 1 *There is a*$\beta_{g,b}$*-orthogonal direct sum decomposition*:

(20) $$\Omega (M;E)≅\Omega_{\Delta_{B}}(M;E)(\lambda )⊕\Omega_{\Delta_{B}}(M;E){\left(\lambda \right)}^{c}.$$

*If*$\lambda ,\mu \in \mathrm{Spec}(\Delta_{B})$*with*λ≠μ*, then*$\Omega_{\Delta_{B}}(M;E)(\mu ){⊥}_{\beta }\Omega_{\Delta_{B}}(M;E)(\lambda )$*. In particular,*$\beta_{g,b}$*restricts to each of these subspaces as a nondegenerate symmetric bilinear form. Furthermore, with the notation in Section*1.3*, there is a*$\beta_{g,b}$*-orthogonal direct sum decomposition*

(21) $$\Omega (M;E){|}_{B_{-}^{0}}≅\Omega_{\Delta_{B}}(M;E)(\lambda )⊕\Omega_{\Delta_{B}}(M;E){\left(\lambda \right)}^{c}{|}_{B_{-}^{0}},$$

*which is invariant under*$d_{E}$*.*

Proof Remark that $\Omega_{\Delta_{B}}(M;E)(\lambda )=P_{\Delta_{B}}(\lambda )(\Omega (M;E))$. Therefore the decomposition in (20) follows from the direct sum decomposition of $L^{2}(M;E)$ stated in Lemma 3. We show that $\Omega_{\Delta_{B}}(M;E)(\lambda )$ is $\beta_{g,b}$-orthogonal to $\Omega_{\Delta_{B}}(M;E){\left(\lambda \right)}^{c}$, by taking $v\in \Omega_{\Delta_{B}}(M;E)(\lambda )$ and $w\in \Omega_{\Delta_{B}}(M;E){\left(\lambda \right)}^{c}$ and noticing that

$$\beta_{g,b}(v,w)=\beta_{g,b}\left(P_{\Delta_{B}}(\lambda )v,w\right)=\beta_{g,b}\left(v,P_{\Delta_{B}}(\lambda )w\right)=0,$$

where the second equality above follows from Lemma 5 and the last one is true because *w* is in the image of the complementary projection of $P_{\Delta_{B}}(\lambda )$. Since $\Omega_{\Delta_{B}}(M;E)(\lambda )$ is contained in the space $\Omega (M;E){|}_{B_{-}^{0}}$, the decomposition in (20) implies directness and $\beta_{g,b}$-orthogonality for the one in (21). By Lemma 4, $\Omega_{\Delta_{B}}(M;E)(\lambda )$ is invariant under both $d_{E}$ and $d_{E,g,b}^{♯}$. But, the space $d_{E}(\Omega_{\Delta_{B}}(M;E){\left(\lambda \right)}^{c}{|}_{B_{-}^{0}})$ is contained in $\Omega_{\Delta_{B}}(M;E){\left(\lambda \right)}^{c}{|}_{B_{-}^{0}}$ as well, as it can be checked by using the Greenʼs formulas from Lemma 3, that $d_{E,g,b}^{♯}$ leaves invariant $\Omega_{\Delta_{B}}(M;E)(\lambda )$ and $\beta_{g,b}$-orthogonality of (20).  □

Corollary 1 *For* $\lambda \in \mathrm{Spec}(\Delta_{B})$ *and with the notation in* (8) *, consider the space* $\Omega_{\Delta_{B}}(M;E){\left(\lambda \right)}^{c}{|}_{B^{0}}$ *. Then, the spaces* $d_{E}(\Omega_{\Delta_{B}}(M;E){\left(\lambda \right)}^{c}{|}_{B^{0}})$ *and* $d_{E,g,b}^{♯}(\Omega_{\Delta_{B}}(M;E){\left(\lambda \right)}^{c}{|}_{B^{0}})$ *are* $\beta_{g,b}$ *-orthogonal to* $\Omega_{\Delta_{B}}(M;E)(\lambda )$ *.*

Proof If $u\in \Omega_{\Delta_{B}}(M;E)(\lambda )$ and $v\in \Omega_{\Delta_{B}}(M;E){\left(\lambda \right)}^{c}{|}_{B^{0}}$, then, by using Lemma 1, invariance of $\Omega_{\Delta_{B}}(M;E)(\lambda )$ under $d_{E,g,b}^{♯}$ (see also Lemma 4 and Proposition 1 above), we have $\beta_{g,b}(u,d_{E}\;v)=\beta_{g,b}(d_{E,g,b}^{♯}\;u,v)=0$. The proof for $d_{E,g,b}^{♯}$ is analog.  □

Corollary 2 **Hodge decomposition**

*We have the* $\beta_{g,b}$ *-orthogonal decomposition*

$$\Omega (M;E)≅\Omega_{\Delta_{B}}(M;E)(0)⊕\Delta_{E,g,b}\left(\Omega_{\Delta_{B}}(M;E){\left(0\right)}^{c}{|}_{B}\right).$$

Proof This follows from Proposition 1 and the isomorphism in (19).  □

Compare the following result with [6, Proposition 2.1].

Proposition 2 *The following are*$\beta_{g,b}$*-orthogonal direct sum decompositions*:

(22) $$\Omega (M;E)≅\Omega_{\Delta_{B}}(M;E)(0)⊕d_{E}\left(d_{E,g,b}^{♯}\left(\Omega_{\Delta_{B}}(M;E){\left(0\right)}^{c}{|}_{B}\right)\right)⊕d_{E,g,b}^{♯}\left(d_{E}\left(\Omega_{\Delta_{B}}(M;E){\left(0\right)}^{c}{|}_{B}\right)\right),$$

(23) $$\Omega (M;E){|}_{B_{-}^{0}}≅\Omega_{\Delta_{B}}(M;E)(0)⊕d_{E}\left(\Omega_{\Delta_{B}}(M;E){\left(0\right)}^{c}{|}_{B_{-}^{0}}\right)⊕d_{E,g,b}^{♯}\left(\Omega_{\Delta_{B}}(M;E){\left(0\right)}^{c}{|}_{B}\right),$$

(24) $$\Omega (M;E){|}_{B^{0}}≅\Omega_{\Delta_{B}}(M;E)(0)⊕d_{E}\left(\Omega_{\Delta_{B}}(M;E){\left(0\right)}^{c}{|}_{B}\right)⊕d_{E,g,b}^{♯}\left(\Omega_{\Delta_{B}}(M;E){\left(0\right)}^{c}{|}_{B}\right).$$

*Moreover, the restriction of*$\beta_{g,b}$*to each of the spaces appearing above is nondegenerate.*

Proof We prove (22). From Corollary 2, every u∈Ω(M;E) can be written as $u=u_{0}+d_{E}(d_{E,g,b}^{♯}\;u)+d_{E,g,b}^{♯}(d_{E}\;u)$, with $u_{0}\in \Omega_{\Delta_{B}}(M;E)(0)$ and $u\in \Omega_{\Delta_{B}}(M;E){\left(0\right)}^{c}{|}_{B}$. That

$$d_{E}\left(d_{E,g,b}^{♯}\left(\Omega_{\Delta_{B}}(M;E){\left(0\right)}^{c}{|}_{B}\right)\right){⊥}_{\beta_{g,b}}d_{E,g,b}^{♯}\left(d_{E}\left(\Omega_{\Delta_{B}}(M;E){\left(0\right)}^{c}{|}_{B}\right)\right),$$

follows from Lemma 1 and ${d_{E}}^{2}=0$. To see that (22) is a direct sum, we check that the intersection of the last two spaces on the right of (22) is trivial. So, take $u\in \Omega_{\Delta_{B}}(M;E){\left(0\right)}^{c}$, and suppose there are $v,w\in \Omega_{\Delta_{B}}(M;E){\left(0\right)}^{c}{|}_{B}$ with $u=d_{E}(d_{E,g,b}^{♯}\;v)=d_{E,g,b}^{♯}(d_{E}\;w)$. Remark obviously that $\Delta_{E,g,b}u=0$ but also that $u\in \Omega_{\Delta_{B}}(M;E)(0)$, since

(a) $i_{-}^{⁎}u=d_{E}(i_{-}^{⁎}d_{E,g,b}^{♯}\;v)=0$, as *v* satisfies boundary conditions,
(b) $i_{-}^{⁎}d_{E,g,b}^{♯}\;u=i_{-}^{⁎}d_{E,g,b}^{♯}\;d_{E,g,b}^{♯}\;d_{E}\;v=0$,
(c) $i_{+}^{⁎}{⋆}_{b}u=\pm d_{E}(i_{+}^{⁎}d_{E,g,b}^{♯}\;{⋆}_{b}w)=0$; as *w* satisfies boundary conditions,
(d) $i_{+}^{⁎}d_{E,g,b}^{♯}\;{⋆}_{b}u=\pm i_{+}^{⁎}{⋆}_{b}d_{E}(d_{E}\;d_{E,g,b}^{♯}\;v)=0$;

therefore, from Proposition 1, *u* must vanish, so that the sum in (22) is direct. This decomposition is clearly $\beta_{g,b}$-orthogonal. The decompositions in (23), (24) follow from that in (22), Lemma 1, the isomorphism in (19) and the definition of boundary conditions as we have proceeded to prove the statement (22); we omit the details. Now, since $d_{E}(\Omega_{\Delta_{B}}(M;E){\left(0\right)}^{c}{|}_{B})\subset d_{E}(\Omega_{\Delta_{B}}(M;E){\left(0\right)}^{c}{|}_{B_{-}^{0}})$, directness of decomposition (24) follows from that of (23). To check directness in (23), firstly observe that by Proposition 1 we have $d_{E}(\Omega_{\Delta_{B}}(M;E){\left(0\right)}^{c}{|}_{B_{-}^{0}})\subset \Omega_{\Delta_{B}}(M;E){\left(0\right)}^{c}{|}_{B_{-}^{0}}$ and therefore the intersection of the space $\Omega_{\Delta_{B}}(M;E)(0)$ with $d_{E}(\Omega_{\Delta_{B}}(M;E){\left(0\right)}^{c}{|}_{B_{-}^{0}})$ is trivial. Secondly, from the inclusion $\Omega_{\Delta_{B}}(M;E){\left(0\right)}^{c}{|}_{B}\subset \Omega_{\Delta_{B}}(M;E){\left(0\right)}^{c}{|}_{B^{0}}$, Corollary 1 and Proposition 1, the intersection of $\Omega_{\Delta_{B}}(M;E)(0)$ with the space $d_{E,g,b}^{♯}(\Omega_{\Delta_{B}}(M;E){\left(0\right)}^{c}{|}_{B})$ is also trivial. Thirdly, the intersection between $d_{E}(\Omega_{\Delta_{B}}(M;E){\left(0\right)}^{c}{|}_{B_{-}^{0}})$ and $d_{E,g,b}^{♯}(\Omega_{\Delta_{B}}(M;E){\left(0\right)}^{c}{|}_{B})$ is trivial as well; indeed, if $u\in \Omega_{\Delta_{B}}(M;E){\left(0\right)}^{c}$ with $u=d_{E}\;v$ for certain $v\in \Omega_{\Delta_{B}}(M;E){\left(0\right)}^{c}{|}_{B_{-}^{0}}$ and $u=d_{E,g,b}^{♯}\;w$ for $w\in d_{E,g,b}^{♯}(\Omega_{\Delta_{B}}(M;E){\left(0\right)}^{c}{|}_{B})$, then, it is follows that $u\in \Omega_{\Delta_{B}}(M;E)(0)$, and therefore u=0. Finally, the bilinear form $\beta_{g,b}$ is nondegenerate on each of the spaces appearing in the direct sum decompositions (i), (ii) and (iii). Indeed, on the one hand, $\beta_{g,b}$ is nondegenerate on each of the spaces appearing on the left hand side of the equalities (i), (ii) and (iii), exactly for the same reason as $\beta_{g,b}$ is nondegenerate on $\Omega_{0}(M;E)$, the space of smooth forms compactly supported in the interior of *M*; this follows immediately from the requirement for *b* to be fiberwise nondegenerate on *E*. On the other hand, from Lemma 1, the direct sum decompositions in (22), (23), (24) are $\beta_{g,b}$-orthogonal. Thus, $\beta_{g,b}$ restricts to each space appearing on the right hand side of (22), (23), (24) as a nondegenerate bilinear form as well.  □

### 1.9 Cohomology

Recall the notation suggested in Lemma 1. The space $\Omega (M;E){|}_{B_{-}^{0}}$ endowed with the differential $d_{E}$ is a cochain complex, which computes de-Rham cohomology of *M* relative to $\partial_{-}M$ with coefficients on *E*, see for instance [3]. For $\lambda \in \mathrm{Spec}(\Delta_{B})$, consider $\Omega_{\Delta_{B}}(M;E)(\lambda )$ as a cochain subcomplex of $\Omega (M;E){|}_{B_{-}^{0}}$. From Lemma 3, Lemma 4 and the isomorphism in (19), every generalized eigenspace corresponding to a *non-zero* eigenvalue is acyclic, i.e., $H(\Omega_{\Delta_{B}}(M;E)(\lambda ))=0$ whenever λ≠0. For λ=0, we have the following.

Proposition 3 *The inclusion*$\Omega_{\Delta_{B}}(M;E)(0)↪\Omega (M;E){|}_{B_{-}^{0}}$*induces an isomorphism in cohomology:*$H^{⁎}(\Omega_{\Delta_{B}}(M;E)(0))≅H^{⁎}(M,\partial_{-}M,E)$*.*

Proof Since $\Omega_{\Delta_{B}}(M;E)(0)\subset \Omega (M;E){|}_{B_{-}^{0}}$, the space $\Omega (M;E){|}_{B_{-}^{0}}$ admits a decomposition compatible with the one in Corollary 2 and therefore it decomposes as

$$\Omega (M;E){|}_{B_{-}^{0}}≅{\Omega_{\Delta_{B}}(M;E)(0)⊕\Delta_{E,g,b}\left(\Omega_{\Delta_{B}}(M;E){\left(0\right)}^{c}{|}_{B}\right)|}_{B_{-}^{0}},$$

where $\Delta_{E,g,b}(\Omega_{\Delta_{B}}(M;E){\left(0\right)}^{c}{|}_{B}){|}_{B_{-}^{0}}$ is also a cochain subcomplex, because of Proposition 1 and that $\Omega (M;E){|}_{B_{-}^{0}}$ is invariant under the action of $d_{E}$. Thus the assertion is true, if the corresponding cohomology groups vanish; that is, if every closed form *w* in $\Delta_{E,g,b}(\Omega_{\Delta_{B}}(M;E){\left(0\right)}^{c}{|}_{B}){|}_{B_{-}^{0}}$ is also exact. By Proposition 2. (23), there exist $w_{1}\in \Omega_{\Delta_{B}}(M;E){\left(0\right)}^{c}{|}_{B_{-}^{0}}$ and $w_{2}\in \Omega_{\Delta_{B}}(M;E){\left(0\right)}^{c}{|}_{B}$ such that $w=d_{E}\;w_{1}+d_{E,g,b}^{♯}\;w_{2}$. First, we claim that $\beta_{g,b}(d_{E,g,b}^{♯}\;w_{2},v_{1})=0$, for all $v_{1}\in \Omega_{\Delta_{B}}(M;E){\left(0\right)}^{c}{|}_{B^{0}}$, see (8); indeed, from Proposition 2.(22), there exist $v_{2},u_{2}\in \Omega_{\Delta_{B}}(M;E){\left(0\right)}^{c}{|}_{B}$, such that $v_{1}=d_{E}\;v_{2}+d_{E,g,b}^{♯}\;u_{2}$ and hence $\beta_{g,b}(d_{E,g,b}^{♯}\;w_{2},d_{E}\;v_{2}+d_{E,g,b}^{♯}\;u_{2})=0$, where we have used that $d_{E,g,b}^{♯}\;w_{2}$, $d_{E}\;v_{2}$ and $d_{E,g,b}^{♯}\;u_{2}\in \Omega_{\Delta_{B}}(M;E){\left(0\right)}^{c}{|}_{B^{0}}$, Lemma 1, ${\left(d_{E,g,b}^{♯}\right)}^{2}=0$ and that $\beta_{g,b}(d_{E}\;d_{E,g,b}^{♯}\;w_{2},u_{2})$ vanishes, because *w* being close implies $d_{E}\;d_{E,g,b}^{♯}\;w_{2}=0$. Finally, since $d_{E,g,b}^{♯}\;w_{2}$ belongs to $\Omega_{\Delta_{B}}(M;E){\left(0\right)}^{c}{|}_{B^{0}}$ as well, and that $\beta_{g,b}$ restricted to this subspace is also nondegenerate, see Proposition 2, from the claim above, we have $d_{E,g,b}^{♯}\;w_{2}=0$. That is, *w* is exact in $\Delta_{E,g,b}(\Omega_{\Delta_{B}}(M;E){\left(0\right)}^{c}{|}_{B}){|}_{B_{-}^{0}}$. □

## 2 Heat trace asymptotic expansion and anomaly formulas

### 2.1 Heat trace asymptotics for an elliptic boundary value problem

Let (D,B) be a boundary value problem, where D is an operator of Laplace type and B is a boundary operator specifying absolute/relative boundary conditions, (or more generally *mixed boundary conditions*, see [13]) and denote by $D_{B}$ its $L^{2}$-realization, see Section 1.6. Then, by [13, Theorem 1.4.5], for t>0 the heat kernel $\exp(-tD_{B})$ is a smoothing operator, of trace class in $L^{2}$-norm and for t→0, there is a complete asymptotic expansion:

$${\mathrm{Tr}}_{L^{2}}\left(\psi \exp(-tD_{B})\right)∼\sum_{n=0}^{\infty }a_{n}(\psi ,D,B)t^{(n-m)/2},$$

where *ψ* is a bundle endomorphism. The coefficients $a_{n}(\psi ,D,B)$, the *heat trace asymptotic coefficients associated to ψ and the boundary value problem* (D,B), are given by the formula

(25) $$a_{n}(\psi ,D,B)=\int_{M}\mathrm{Tr}\left(\psi \cdot e_{n}(D)\right){\mathrm{vol}}_{g}(M)+\sum_{k=0}^{n-1}\int_{\partial M}\mathrm{Tr}\left({\nabla_{\varsigma_{\mathrm{in}}}}^{k}\psi \cdot e_{n,k}(D,B)\right){\mathrm{vol}}_{g}(\partial M),$$

where ${\nabla_{\varsigma_{\mathrm{in}}}}^{k}$ denotes the *k*-covariant derivative along the inwards pointing geodesic unit vector field normal to ∂*M*, computed with respect to the Levi-Cività connection on $\Lambda^{⁎}(T^{⁎}M)$ and an auxiliary connection on the bundle. The quantities $e_{n}(x,D)$ and $e_{n,k}(y,D,B)$ in (25) are invariant endomorphism-valued forms locally computable as polynomials in the jets of the symbol of D and B, see [14], [22], [23], [24]. By using Weylʼs theory of invariants, these endomorphism invariants can be expressible as universal polynomials in locally computable tensorial objects, see [13, Sections 1.7 and 1.8] (see also [12, Sections 1.7, 1.9 and 4.8]) and [13, Section 3.1.8].

We are interested in the *coefficient of the constant term* in the heat asymptotic expansion in (25) corresponding to n=dim(M)=m, which in accord with the notation in [2], we denote by

(26) $$\underset{t\to 0}{\mathrm{LIM}}\left({\mathrm{Tr}}_{L^{2}}\left(\psi \exp(-tD_{B})\right)\right):=a_{m}(\psi ,D,B).$$

### 2.2 Heat trace asymptotics for the Hermitian Laplacian

Brüning and Ma studied in [8] the Hermitian Laplacian on a manifold with boundary under absolute boundary conditions and obtained anomaly formulas for the associated Ray–Singer analytic metric. They do so by computing the coefficient of the constant term in certain heat trace asymptotic expansion associated to the Hermitian boundary value problem.

Proposition 4 below is basically due to the work by Brüning and Ma in [8]. In order to read its statement, we need certain characteristic forms on *M* and ∂*M*. The forms defined on *M*, already appearing in the anomaly formulas for the torsion in the situation without boundary, are the Euler form $e(M,g)\in \Omega^{m}(M;\Theta_{M})$, associated to the metric *g*, and secondary forms of Chern–Simons type $\tilde{e}(M,g,g^{\prime })\in \Omega^{m-1}(M;\Theta_{M})$ associated to two (smoothly connected) Riemannian metrics *g* and $g^{\prime }$. The forms defined on ∂*M*, already defined by Brüning and Ma, are on the one hand $e_{b}(\partial M,g)$ and $B(\partial M,g)\in \Omega^{m-1}(\partial M;\Theta_{M})$, see [8, expression (1.17), p. 775] and on the other certain Chern–Simons forms ${\tilde{e}}_{b}(\partial M,g,g^{\prime })\in \Omega^{m-2}(\partial M;\Theta_{M})$, see [8, expression (1.45), p. 780]. For the sake of completeness, we recall in Appendix A, how these characteristic forms were constructed in [8].

Proposition 4 **Brüning–Ma**

*Recall the remarks and the notation from Section* 1.4 *. Let* (M,∂M,∅) *be a compact Riemannian bordism. Consider* ${\left[\Delta ,B\right]}_{\left(M,\partial M,\emptyset \right)}^{E,g,h}$ *the Hermitian boundary value problem and denote by* $\Delta_{\mathrm{abs},h}$ *its* $L^{2}$ *-realization. For* ϕ∈Γ(M,End(E)) *we have*

(27) $$\underset{t\to 0}{\mathrm{LIM}}\left(S\mathrm{Tr}\left(\phi \exp(-t\Delta_{\mathrm{abs},h})\right)\right)=\int_{M}\mathrm{Tr}(\phi )e(M,g)-{\left(-1\right)}^{m}\int_{\partial M}i^{⁎}\mathrm{Tr}(\phi )e_{b}(\partial M,g),$$

*where* STr *stands for supertrace. Moreover, for* ξ∈Γ(M,End(TM)) *, a symmetric endomorphism with respect to g, and* $D^{⁎}\xi \in \Gamma (M,\mathrm{End}(\Lambda^{⁎}T^{⁎}M))$ *, its extension as a derivation on* $\Lambda^{⁎}(T^{⁎}M)$ *, set*

(28) $$\Psi :=D^{⁎}\xi -\frac{1}{2}\mathrm{Tr}(\xi ).$$

*If* τ∈R *is taken small enough so that* g+τgξ *is a nondegenerate symmetric metric on TM, then*

(29) $$\underset{t\to 0}{\mathrm{LIM}}\left(S\mathrm{Tr}\left(-\Psi \exp(-t\Delta_{\mathrm{abs},h})\right)\right)=-2\int_{M}{\frac{\partial }{\partial \tau }|}_{\tau =0}\tilde{e}(M,g,g+\tau g\xi )\wedge \omega \left(\nabla^{E},h\right)+2\int_{\partial M}-{\frac{\partial }{\partial \tau }|}_{\tau =0}{\tilde{e}}_{b}(\partial M,g,g+\tau g\xi )\wedge i^{⁎}\omega \left(\nabla^{E},h\right)+\mathrm{rank}(E)\int_{\partial M}{\frac{\partial }{\partial \tau }|}_{\tau =0}B(\partial M,g+\tau g\xi ),$$

*where* $\omega (\nabla^{E},h):=-\frac{1}{2}\mathrm{Tr}(h^{-1}\nabla^{E}h)$ *is a real valued closed one-form.*

Proof We prove formula (27). First, each ϕ∈Γ(M,End(E)) can be uniquely written as $\phi =\phi^{\mathrm{re}}+i\phi^{\mathrm{im}}$ where $\phi^{\mathrm{re}},\phi^{\mathrm{im}}$ are selfadjoint elements. Thus, it is enough to prove (27) for *ϕ* selfadjoint. First, suppose that $\phi_{u}:=h_{u}^{-1}\frac{\partial h_{u}}{\partial u}\in \Gamma (M,\mathrm{End}(E))$, where $h_{u}$ is a smooth one real parameter family of Hermitian forms on *E* with $h_{0}=h$. Then, (27) exactly is the infinitesimal version of Brüning and Maʼs formulas, see [8, Theorem 4.6] and [8, expression (5.72)]. Next, suppose ϕ∈Γ(M,End(E)) to be an arbitrary selfadjoint element. Then, for *u* small enough, the family $h_{u}:=h+uh\phi$ is a smooth family of Hermitian forms on *E* and $h_{u}^{-1}\frac{\partial h_{u}}{\partial u}=h_{u}^{-1}h\phi$ defines a smooth family of selfadjoint elements in Γ(M,End(E)). Therefore, we apply Brüning and Maʼs formulas for $h_{0}^{-1}(\frac{\partial h_{u}}{\partial u}{|}_{u=0})=\phi$ so that the proof of (27) is complete. We now prove (29). Let $g_{u}$ be a smooth family of Riemannian metrics on *TM* with $g_{0}=g$ and denote by ${⋆}_{u}$ the Hodge ⋆-operator corresponding to $g_{u}$. First, consider the case where $\xi_{u}:=g_{u}^{-1}\frac{\partial g_{u}}{\partial u}\in \Gamma (M;\mathrm{End}(TM))$ so that, by (28), we obtain $\Psi_{u}=D^{⁎}(g_{u}^{-1}\frac{\partial g_{u}}{\partial u})-\frac{1}{2}\mathrm{Tr}(g_{u}^{-1}\frac{\partial g_{u}}{\partial u})=-{⋆}_{u}^{-1}\frac{\partial {⋆}_{u}}{\partial u}$, see [7, Proposition 4.15], considered as a smooth family in $\Gamma (M,\mathrm{End}(\Lambda^{⁎}T^{⁎}M))$. Then, (29) is the infinitesimal version of Brüning and Maʼs formulas, see [8, Theorem 4.6] and [8, expressions (5.74) and (5.75)]. In the general case, take a symmetric ξ∈Γ(M;End(TM)). Then, for *u* small enough the formula $g_{u}:=g+ug\xi$ defines a smooth family of nondegenerate metrics on *TM* and hence $g_{u}^{-1}\frac{\partial g_{u}}{\partial u}=g_{u}^{-1}g\xi$ a smooth family of symmetric elements in Γ(M,End(TM)). Hence we obtain a smooth family of symmetric endomorphisms $-{⋆}_{u}^{-1}\frac{\partial {⋆}_{u}}{\partial u}$ in $\Gamma (M,\mathrm{End}(\Lambda^{⁎}T^{⁎}M))$, for which we can use again Brüning and Maʼs formulas. In particular, they must hold for u=0 for which we have $g_{0}^{-1}(\frac{\partial g_{u}}{\partial u}{|}_{u=0})=\xi$, so that $\Psi_{0}=D^{⁎}(\xi )-\frac{1}{2}\mathrm{Tr}(\xi )=-{⋆}_{0}^{-1}(\frac{\partial {⋆}_{u}}{\partial u}{|}_{u=0})$. That is, (29) holds.  □

Lemma 6 *Let* ${\bar{E}}^{\prime }$ *be the dual of the complex conjugated vector bundle of E, endowed with the dual flat connection and dual Hermitian form to those on E. Consider the compact Riemannian bordisms* (M,∅,∂M) *together with its dual* ${\left(M,\emptyset ,\partial M\right)}^{\prime }:=(M,\partial M,\emptyset )$ *. Let* $\Delta_{\mathrm{rel},h}$ *be the* $L^{2}$ *-realization associated to the Hermitian boundary value problem* ${\left[\Delta ,B\right]}_{\left(M,\emptyset ,\partial M\right)}^{E,g,h}$ *and* $\Delta_{\mathrm{abs},h^{\prime }}^{\prime }$ *the one associated to* ${\left[\Delta ,B\right]}_{{\left(M,\emptyset ,\partial M\right)}^{\prime }}^{{\bar{E}}^{\prime }⊗\Theta_{M},g,h^{\prime }}$ *. If ϕ, ξ and Ψ are as in* Proposition 4 *, then*

(30) $$\underset{t\to 0}{\mathrm{LIM}}\left(S\mathrm{Tr}\left(\phi \exp(-t\Delta_{\mathrm{rel},h})\right)\right)={\left(-1\right)}^{m}\underset{t\to 0}{\mathrm{LIM}}\left(S\mathrm{Tr}\left(\phi^{⁎}\exp-t\Delta_{\mathrm{abs},h^{\prime }}^{\prime }\right)\right),$$

*where* $\phi^{⁎}:=h\phi h^{-1}$ *, and*

(31) $$\underset{t\to 0}{\mathrm{LIM}}S\mathrm{Tr}\left(\Psi \exp(-t\Delta_{\mathrm{rel},h})\right)={\left(-1\right)}^{m+1}\underset{t\to 0}{\mathrm{LIM}}S\mathrm{Tr}\left(\Psi \exp\left(-t\Delta_{\mathrm{abs},h^{\prime }}^{\prime }\right)\right).$$

Proof Consider $h\in \Omega^{0}(M;\mathrm{End}(E,{\bar{E}}^{\prime }))$ the complex vector bundle isomorphism between *E* and ${\bar{E}}^{\prime }$ provided by the Hermitian metric on *E* (see for instance [3, p. 286]), and its covariant derivative $\nabla^{E}h\in \Omega^{1}(M;\mathrm{End}(E,{\bar{E}}^{\prime }))$ computed by using the induced connection on $\mathrm{End}(E,{\bar{E}}^{\prime })$. With the Hermitian metric on *E* and the Riemannian metric on *M*, we have a *complex-linear* isomorphism ${⋆}_{h}:=⋆⊗h:\Omega (M;E)\to \Omega (M;{\bar{E}}^{\prime }⊗\Theta_{M})$, which is used to define

$$d_{E,g,h}^{⁎}:={\left(-1\right)}^{q}{⋆}_{h}^{-1}\;d_{{\bar{E}}^{\prime }⊗\Theta_{M}}{⋆}_{h}:\Omega^{q}(M;E)\to \Omega^{q-1}(M;E);$$

being the formal adjoint to $d_{E}$ with respect to the Hermitian product on Ω(M;E). Remark here that the formula

$$d_{{\bar{E}}^{\prime }⊗\Theta_{M}}\;d_{{\bar{E}}^{\prime }⊗\Theta_{M},g,h^{\prime }}^{⁎}{⋆}_{h}={⋆}_{h}\;d_{E,g,h}^{⁎}d_{E}$$

holds and therefore

$${⋆}_{h}\Delta_{E,g,h}=\Delta_{{\bar{E}}^{\prime }⊗\Theta_{M},g,h^{\prime }}{⋆}_{h}.$$

As in Section 1.4, the operator ${⋆}_{h}$ intertwines *E*-valued forms satisfying relative (resp. absolute) boundary conditions with ${\bar{E}}^{\prime }$-valued forms satisfying absolute (resp. relative) boundary conditions. That is,

(32) $$\Delta_{\mathrm{rel},h}={⋆}_{h}^{-1}\Delta_{\mathrm{abs},h^{\prime }}^{\prime }{⋆}_{h}$$

and therefore $\phi \exp(-t\Delta_{\mathrm{rel},h})={⋆}_{h}^{-1}\phi^{⁎}\exp(-t\Delta_{\mathrm{abs},h^{\prime }}^{\prime }){⋆}_{h}$, where $\phi^{⁎}:=h\phi h^{\prime }$. Thus, since the supertrace vanishes on supercommutators of graded complex-linear operators and the degree of ${⋆}_{h,q}$ is m−q, we obtain the formula

$$S\mathrm{Tr}\left(\phi \exp(-t\Delta_{\mathrm{rel},h})\right)={\left(-1\right)}^{m}S\mathrm{Tr}\left(\phi^{⁎}\exp\left(-t\Delta_{\mathrm{abs},h^{\prime }}^{\prime }\right)\right)$$

and hence (30). We now turn to formula (31). First, remark that

(33) $${⋆}_{q}\left(D^{⁎}\xi -\frac{1}{2}\mathrm{Tr}(\xi )\right){⋆}_{q}^{-1}=-D^{⁎}\xi +\frac{1}{2}\mathrm{Tr}(\xi ).$$

We prove (33), by pointwise computing ${⋆}_{q}D^{⁎}\xi {⋆}_{q}^{-1}$. Since *ξ* is a symmetric complex endomorphism of $T_{x}M$, we may choose an orthonormal frame ${\left\{e_{i}\right\}}_{1}^{m}$ such that $\xi e_{i}=\lambda_{i}e_{i}$. Then, for ${\left\{e^{i_{1}}\wedge \cdots \wedge e^{i_{q}}\right\}}_{1\leq i_{1}<\cdots <i_{q}\leq m}$ a positive definite oriented frame for $\Lambda^{q}T_{x}^{⁎}M$, the Hodge ⋆-operator is given by ${⋆}_{q}(e^{i_{1}}\wedge \cdots \wedge e^{i_{q}})=e^{j_{1}}\wedge \cdots \wedge e^{j_{m-q}}\in \Lambda^{m-q}T_{x}^{⁎}M$, where the ordered indices $\left(j_{1},\ldots ,j_{m-q}):=(1,\ldots ,\hat{i_{1}},\ldots ,\hat{i_{q}},\ldots ,m\right)$ with $1\leq j_{1}<\cdots <j_{m-q}\leq m$, are obtained as the unique possible choice of ordered indices complementary to $\leq i_{1}<\cdots <i_{q}$. Therefore

$${⋆}_{q}D^{⁎}\xi {⋆}_{q}^{-1}\left(e^{j_{1}}\wedge \cdots \wedge e^{j_{m-q}}\right)={⋆}_{q}D^{⁎}\xi \left(e^{i_{1}}\wedge \cdots \wedge e^{i_{q}}\right)={⋆}_{q}\sum_{l=1}^{q}\left(e^{i_{1}}\wedge \cdots \wedge \xi \left(e^{i_{l}}\right)\wedge \cdots \wedge e^{i_{q}}\right)={⋆}_{q}\sum_{l=1}^{q}\lambda_{i_{l}}\left(e^{i_{1}}\wedge \cdots \wedge e^{i_{l}}\wedge \cdots \wedge e^{i_{q}}\right)=\sum_{l=1}^{q}\lambda_{i_{l}}\left(e^{j_{1}}\wedge \cdots \wedge e^{j_{m-q}}\right)=\sum_{l=1}^{m}\lambda_{i_{l}}\left(e^{j_{1}}\wedge \cdots \wedge e^{j_{m-q}}\right)-\sum_{l=1}^{m-q}\lambda_{j_{l}}\left(e^{j_{1}}\wedge \cdots \wedge e^{j_{m-q}}\right)=\sum_{l=1}^{m}\lambda_{i_{l}}\left(e^{j_{1}}\wedge \cdots \wedge e^{j_{m-q}}\right)-\sum_{l=1}^{m-q}\left(e^{j_{1}}\wedge \cdots \wedge \lambda_{j_{l}}e^{j_{l}}\wedge \cdots \wedge e^{j_{m-q}}\right)=\left(\mathrm{Tr}\xi -D^{⁎}\xi \right)\left(e^{j_{1}}\wedge \cdots \wedge e^{j_{m-q}}\right)$$

and we obtain (33), which in turn allows us to conclude

(34) $$\Psi {\left({⋆}_{q}⊗h\right)}^{-1}=\left(\left(D^{⁎}\xi -\frac{1}{2}\mathrm{Tr}(\xi )\right)⊗1\right){\left({⋆}_{q}⊗h\right)}^{-1}={\left({⋆}_{q}⊗h\right)}^{-1}\left(\left({⋆}_{q}\left(D^{⁎}\xi -\frac{1}{2}\mathrm{Tr}(\xi )\right){⋆}_{q}^{-1}\right)⊗1\right)=-{\left({⋆}_{q}⊗h\right)}^{-1}\left(\left(D^{⁎}\xi -\frac{1}{2}\mathrm{Tr}(\xi )\right)⊗1\right)=-{\left({⋆}_{q}⊗h\right)}^{-1}\Psi .$$

Finally, we use (34) to pass to the complex conjugated; hence with (32) and duality between these boundary value problems we obtain

$$\Psi \exp(-t\Delta_{\mathrm{rel},h})=\Psi {⋆}_{h}^{-1}\exp\left(-t\Delta_{\mathrm{abs},h^{\prime }}^{\prime }\right){⋆}_{h}=-{⋆}_{h}^{-1}\Psi \exp\left(-t\Delta_{\mathrm{abs},h^{\prime }}^{\prime }\right){⋆}_{h}$$

thus, as for (30), we have

$$S\mathrm{Tr}\left(\Psi \exp(-t\Delta_{\mathrm{rel},h})\right)=-{\left(-1\right)}^{m}S\mathrm{Tr}\left(\Psi \exp\left(-t\Delta_{\mathrm{abs},h^{\prime }}^{\prime }\right)\right).\;□$$

Proposition 5 *For the Riemannian bordism* (M,∅,∂M) *, consider the Hermitian boundary value problem* ${\left[\Delta ,B\right]}_{\left(M,\emptyset ,\partial M\right)}^{E,g,h}$ *with its* $L^{2}$ *-realization denoted by* $\Delta_{\mathrm{rel},h}$ *. If ϕ, ξ and Ψ are as in* Proposition 4 *, then*

$$\underset{t\to 0}{\mathrm{LIM}}\left(S\mathrm{Tr}\left(\phi \exp(-t\Delta_{\mathrm{rel},h})\right)\right)=\int_{M}\mathrm{Tr}(\phi )e(M,g)-\int_{\partial M}i^{⁎}\mathrm{Tr}(\phi )e_{b}(\partial M,g)$$

*and*

$$\underset{t\to 0}{\mathrm{LIM}}\left(S\mathrm{Tr}\left(-\Psi \exp(-t\Delta_{\mathrm{rel},h})\right)\right)=-2\int_{M}{\frac{\partial }{\partial \tau }|}_{\tau =0}\tilde{e}(M,g,g+\tau g\xi )\wedge \omega \left(\nabla^{E},h\right)+2{\left(-1\right)}^{m+1}\int_{\partial M}{\frac{\partial }{\partial \tau }|}_{\tau =0}{\tilde{e}}_{b}(\partial M,g,g+\tau g\xi )\wedge i^{⁎}\omega \left(\nabla^{E},h\right)+{\left(-1\right)}^{m+1}\mathrm{rank}(E)\int_{\partial M}{\frac{\partial }{\partial \tau }|}_{\tau =0}B(\partial M,g+\tau g\xi ).$$

Proof A form $w\in \Omega^{⁎}(M;E)$ satisfies relative boundary conditions if and only if the smooth form ${⋆}_{h}w\in \Omega^{m-⁎}(M;{\bar{E}}^{\prime }⊗\Theta_{M})$ satisfies absolute boundary conditions on ∂*M*. Hence, the first formula in the statement follows from formula (30) in Lemma 6, and the results from Brüning and Ma for the Hermitian Laplacian stated in Proposition 4. The second formula follows from formula (31) in Lemma 6, Proposition 4 and $\omega (\nabla^{E},h)=-\omega (\nabla^{E^{\prime }},h^{\prime })$, see for instance [4, Section 2.4].  □

Lemma 7 *For* (M,∂M,∅) *,* (M,∅,∂M) *and* $\left(M,\partial_{+}M,\partial_{-}M\right)$ *let us consider* ${\left[\Delta ,B\right]}_{\left(M,\partial M,\emptyset \right)}^{E,g,h}$ *,* ${\left[\Delta ,B\right]}_{\left(M,\emptyset ,\partial M\right)}^{E,g,h}$ *and* ${\left[\Delta ,B\right]}_{\left(M,\partial_{+}M,\partial_{-}M\right)}^{E,g,h}$ *the corresponding Hermitian boundary value problems, together with their* $L^{2}$ *-realizations* $\Delta_{\mathrm{abs},h}$ *,* $\Delta_{\mathrm{rel},h}$ *and* $\Delta_{B,h}$ *, respectively. Let* $\psi_{\pm }\in \Gamma (M;\mathrm{End}(\Lambda^{⁎}(T^{⁎}M)⊗E))$ *be chosen in such a way that* $\mathrm{supp}(\psi_{\pm })\cap \partial_{\mp }M=\emptyset$ *, then*

$$\underset{t\to 0}{\mathrm{LIM}}\left(S\mathrm{Tr}\left(\psi_{+}\exp(-t\Delta_{B,h})\right)\right)=\underset{t\to 0}{\mathrm{LIM}}\left(S\mathrm{Tr}\left(\psi_{+}\exp(-t\Delta_{\mathrm{abs},h})\right)\right),$$

$$\underset{t\to 0}{\mathrm{LIM}}\left(S\mathrm{Tr}\left(\psi_{-}\exp(-t\Delta_{B,h})\right)\right)=\underset{t\to 0}{\mathrm{LIM}}\left(S\mathrm{Tr}\left(\psi_{-}\exp(-t\Delta_{\mathrm{rel},h})\right)\right).$$

Proof This is an immediate consequence of $\partial_{+}M$ and $\partial_{-}M$ being mutually disjoint and that the coefficients in the heat kernel asymptotic expansion are computable as universal polynomials in terms of finite order derivatives of the symbols expressed in local coordinates around each point of *M*, see Section 2.1.  □

Theorem 1 *For* $\left(M,\partial_{+}M,\partial_{-}M\right)$ *, consider the Hermitian boundary value problem* ${\left[\Delta ,B\right]}_{\left(M,\partial_{+}M,\partial_{-}M\right)}^{E,g,h}$ *with its corresponding* $L^{2}$ *-realization* $\Delta_{B,h}$ *. If ϕ, ξ and Ψ are as in* Proposition 4 *, then*

$$\underset{t\to 0}{\mathrm{LIM}}\left(S\mathrm{Tr}\left(\phi \exp(-t\Delta_{B,h})\right)\right)=\int_{M}\mathrm{Tr}(\phi )e(M,g)+{\left(-1\right)}^{m-1}\int_{\partial_{+}M}\mathrm{Tr}(\phi )i_{+}^{⁎}e_{b}(\partial M,g)-\int_{\partial_{-}M}\mathrm{Tr}(\phi )i_{-}^{⁎}e_{b}(\partial M,g)$$

*and*

$$\underset{t\to 0}{\mathrm{LIM}}\left(S\mathrm{Tr}\left(-\Psi \exp(-t\Delta_{B,h})\right)\right)=-2\int_{M}{\frac{\partial }{\partial \tau }|}_{\tau =0}\tilde{e}(M,g,g+\tau g\xi )\wedge \omega \left(\nabla^{E},h\right)-2\int_{\partial_{+}M}{\frac{\partial }{\partial \tau }|}_{\tau =0}i_{+}^{⁎}{\tilde{e}}_{b}(\partial M,g,g+\tau g\xi )\wedge \omega \left(\nabla^{E},h\right)+\mathrm{rank}(E)\int_{\partial_{+}M}{\frac{\partial }{\partial \tau }|}_{\tau =0}i_{+}^{⁎}B(\partial M,g+\tau g\xi )-2{\left(-1\right)}^{m}\int_{\partial_{-}M}{\frac{\partial }{\partial \tau }|}_{\tau =0}i_{-}^{⁎}{\tilde{e}}_{b}(\partial M,g,g+\tau g\xi )\wedge \omega \left(\nabla^{E},h\right)+{\left(-1\right)}^{m+1}\mathrm{rank}(E)\int_{\partial_{-}M}{\frac{\partial }{\partial \tau }|}_{\tau =0}i_{-}^{⁎}B(\partial M,g+\tau g\xi ).$$

Proof This follows from Proposition 4 (Brüning and Ma), Proposition 5 and Lemma 7. More recently, Brüning and Ma gave also a proof of this statement, see [9, Theorem 3.2], based on the methods developed in [8].  □

### 2.3 Involutions, bilinear and Hermitian forms

We fix a Hermitian structure compatible with the bilinear one as follows. Since *E* is endowed with a bilinear form *b*, there exists an anti-linear involution *ν* on *E* satisfying

(35) $$\bar{b(\nu e_{1},\nu e_{2})}=b(e_{1},e_{2})\;\text{and}\;b(\nu e,e)>0\;\text{for all }e_{1},e_{2},\;e\in E\text{ with }e\neq 0,$$

see for instance the proof of [4, Theorem 5.10]. In this way, we obtain a (positive definite) Hermitian form on *E* given by

(36) $$h(e_{1},e_{2}):=b(e_{1},\nu e_{2}).$$

Remark that $\nabla^{E}\nu =0$ is not required so that

$$h^{-1}\left(\nabla^{E}h\right)=\nu^{-1}\left(b^{-1}\left(\nabla^{E}b\right)\right)\nu +\nu^{-1}\left(\nabla^{E}\nu \right).$$

Therefore, this yields a Hermitian form on Ω(M;E) compatible with $\beta_{g,b}$ in the sense that ${《v,w》}_{g,h}=\beta_{g,b}(v,\nu w)$. for v,w∈Ω(M;E). In [26] and [25], given a bilinear form *b*, this involution has been exploited to study the bilinear Laplacian in terms of the Hermitian one associated to the compatible Hermitian form in (36), in both cases with and without boundary. However, our approach is a little different since we do not use a Hermitian form globally compatible with $\beta_{g,b}$ on Ω(M;E), but instead a local compatibility only, see Section 2.4 below.

We now study the situation where *ν* is parallel with respect to $\nabla^{E}$.

Lemma 8 *Let us consider*$\left(M,\partial_{+}M,\partial_{-}M\right)$*the compact Riemannian bordism together with the complex flat vector bundle E as above. Suppose E admits a nondegenerate symmetric bilinear form. Moreover, suppose there exists a complex anti-linear involution ν on E, satisfying the conditions in*(35)*and*$\nabla^{E}\nu =0$*. Let h be the* (*positive definite*) *Hermitian form on E compatible with b defined by* (36)*. Then,* $\Delta_{E,g,b}=\Delta_{E,g,h}$ *and* $B_{E,g,b}=B_{E,g,h}$*.*

Proof Consider ${《\cdot ,\cdot 》}_{g,h}$ the Hermitian product on Ω(M;E), compatible with the bilinear form, and $d_{E,g,h}^{⁎}$, the formal adjoint to $d_{E}$ with respect to this product, which in terms of the Hodge ⋆-operator can be written up to a sign as $d_{E,g,h}^{⁎}=\pm {⋆}_{h}^{-1}d_{E}\;{⋆}_{h}$. Remark that $\nabla^{E}\nu =0$ implies that $d_{E}\;\nu =\nu d_{E}$; hence, with ${⋆}_{h}=\nu \circ {⋆}_{b}$, we have

(37) $$d_{E,g,h}^{⁎}=\pm {⋆}_{h}^{-1}d_{E}{⋆}_{h}=\pm {⋆}_{b}^{-1}\nu^{-1}d_{E}\nu {⋆}_{b}=\pm {⋆}_{b}^{-1}d_{E}{⋆}_{b}=d_{E,g,b}^{♯},$$

and therefore the Hermitian and bilinear Laplacians coincide. We turn to the assertion for the corresponding boundary operators. On the one hand, the assertion is clear for ${B_{-}}_{E,g,b}={B_{-}}_{E,g,h}$, because of (37), (7). On the other hand, for a form $v\in \Omega^{p}(M;E)$ and $\iota_{\varsigma_{\mathrm{in}}}$, the interior product with respect to the dual form corresponding to $\varsigma_{\mathrm{in}}$, the identity ${⋆}_{b}^{\partial M}i^{⁎}\iota_{\varsigma_{\mathrm{in}}}v=i^{⁎}{⋆}_{b}^{M}v$ holds; therefore the operator specifying absolute boundary can be written, independently of the Hermitian or bilinear forms, as ${B_{+}}_{E,g,b}^{p}v=(i_{+}^{⁎}\iota_{\varsigma_{\mathrm{in}}}v,{\left(-1\right)}^{p+1}i_{+}^{⁎}\iota_{\varsigma_{\mathrm{in}}}(d_{E}\;v))={B_{+}}_{E,g,h}^{p}v$. That finishes the proof.  □

Lemma 9 *Let*(M,g)*be a compact Riemannian manifold and E a flat complex vector bundle over M. Assume E is endowed with a fiberwise nondegenerate symmetric bilinear form b. For each*x∈M*there exists an open neighborhood U of x in M, a parallel anti-linear involution ν on*$E{|}_{U}$*and a symmetric bilinear form*$\tilde{b}$*on E such that, for*z∈C*, the family of fiberwise symmetric bilinear forms*

(38) $$b_{z}:=b+z\tilde{b},$$

*has the following properties*:

(i) $b_{z}$*is fiberwise nondegenerate for all*z∈C*with*$|z|\leq \sqrt{2}$*,*
(ii) $\bar{b_{s-i}(\nu e_{1},\nu e_{2})}=b_{s-i}(e_{1},e_{2})$*, for all*s∈R*and*$e_{i}\in E{|}_{U}$*,*
(iii) $b_{s-i}(e,\nu e)>0$*for all*s∈R*,*|s|≤1*and*$0\neq e\in E{|}_{U}$*.*

Proof Since flat vector bundles are locally trivial, there exists a neighborhood *V* of *x* and a parallel complex anti-linear involution *ν* on $E{|}_{V}$. Moreover, since *b* is nondegenerate and *ν* an involution, we can assume without loss of generality that *ν* can be chosen to be compatible with *b* at the fiber $E_{x}$ over *x*, such that

$$b_{x}(\nu e_{1},\nu e_{2})=\bar{b_{x}(e_{1},e_{2})}\;\text{for all}\;e_{i}\in E_{x}$$

and

$$b_{x}(\nu e,e)>0\;\text{for all}\;0\neq e\in E_{x}.$$

Consider

bRe(e1,e2):=12(b(e1,e2)+b(νe1,νe2)¯),

bIm(e1,e2):=12i(b(e1,e2)−b(νe1,νe2)¯),

as symmetric bilinear forms on $E{|}_{V}$. In particular, note that by construction

(39) b|V=bRe+ibImwithbIm|Ex=0,

(40) bRe(νe1,νe2)¯=bRe(e1,e2)andbIm(νe1,νe2)¯=bIm(e1,e2),

for all $e_{i}\in E{|}_{V}$. Now, choose an open neighborhood U⊂V of *x* and a compactly supported smooth function λ:V→[0,1] such that $\lambda {|}_{U}=1$. Thus, by extending *λ* by zero to *M*, we set

(41) b˜:=λbIm,

as a globally defined symmetric bilinear form on *E*. Using

bs−i|U=(b+(s−i)b˜)|U=b|U+(s−i)bIm|U=bRe|U+sbIm|U

and (40) we immediately obtain (ii). In turn, (ii) implies

$$\bar{b_{s-i}(\nu e,e)}=b_{s-i}(\nu e,e)$$

and hence $b_{s-i}(\nu e,e)$ is real for all s∈R and $e\in E{|}_{U}$. Finally, by the formula (38) defining $b_{z}$ at *x*, we have bIm|x=0 and therefore

• $b_{z}{|}_{x}$ is nondegenerate,
• $b_{s-i}{|}_{x}(\nu e,e)=b{|}_{x}(\nu e,e)>0$ for all $0\neq e\in E_{x}$,

from which (i) (resp. (iii)) follows by taking $|z|\leq \sqrt{2}$ (resp. |s|≤1) and then choosing the support of *λ* small enough around *x*. □

The following proposition provides the key argument in the proof of Theorem 2 below.

Proposition 6 *Let*${\left[\Delta ,B\right]}_{\left(M,\partial_{+}M,\partial_{-}M\right)}^{E,g,b}$*be the bilinear boundary value problem under absolute and relative boundary conditions on*$\left(M,\partial_{+}M,\partial_{-}M\right)$*. Then, for each*x∈M*, there exist*${\left\{b_{z}\right\}}_{z\in C}$*a family of* fiberwise symmetric bilinear forms *on E, and* ${\left\{h_{s}\right\}}_{s\in R}$ *a family of fiberwise* sesquilinear Hermitian forms *on E such that*

(i) $b_{z}$*is fiberwise nondegenerate for all*z∈C*such that*$|z|\leq \sqrt{2}$*.*
(ii) $h_{s}$*is fiberwise positive definite Hermitian form for*s∈R*with*|s|≤1*.*
(iii) *For each*s∈R*with*|s|≤1*, consider*${\left[\Delta ,\Omega_{B}\right]}_{\left(M,\partial_{+}M,\partial_{-}M\right)}^{E,g,h_{s}}$*the corresponding Hermitian boundary value problem. Then, there exists a neighborhood U of x such that*
  $$\Delta_{E,g,b_{s-i}}{|}_{U}=\Delta_{E,g,h_{s}}{|}_{U}\;\text{and}\;B_{E,g,b_{s-i}}{|}_{U}=B_{E,g,h_{s}}{|}_{U}.$$

Proof By Lemma 9(i), for each x∈M, there exists a globally defined fiberwise symmetric bilinear form $\tilde{b}$ on *E* such that the formula $b_{z}:=b+z\tilde{b}$ in (38) defines a family of fiberwise nondegenerate symmetric bilinear forms on *E*, satisfying the required property in (i). In addition, we know that for each x∈M, there exist an open neighborhood *V* of *x* and a parallel complex anti-linear involution *ν* on $E{|}_{V}$. By Lemma 9(i)–(ii), we also know that we can find U⊂V a small enough open neighborhood of *x*, such that $b_{s-i}$ satisfies the conditions (i) and (ii) on $E{|}_{U}$, for |s|≤1. Hence, by using the formula in (36), we obtain a fiberwise positive definite Hermitian form compatible with $b_{s-i}$ on $E{|}_{U}$ given by $h_{s}^{U}(e_{1},e_{2}):=b_{s-i}(\nu e_{1},e_{2})$. Now we extend $h_{s}^{U}$ to a (positive definite) Hermitian form on *E* as follows. We take $h^{\prime }$ any arbitrary Hermitian form on *E* and consider the finite open covering $\left\{U_{0}^{\prime },U_{1}^{\prime },\ldots ,U_{N}^{\prime }\right\}$ of *M*, with $U_{0}^{\prime }:=U$, together with a subordinate partition of unity ${\left\{f_{j}\right\}}_{U_{j}^{\prime }}$. If $h_{j}^{\prime }:=h^{\prime }{|}_{U_{j}}$, then $h_{s}:=f_{0}h_{s}^{U}+\sum_{j=1}^{N}f_{j}h_{j}^{\prime }$ globally defines a fiberwise positive definite Hermitian form on *E*, as the space of Hermitian forms on *E* is a convex space. This proves (ii). Then, (iii) follows from Lemma 8.  □

### 2.4 Heat trace asymptotics for bilinear boundary value problems

Lemma 10 *Let O be an open connected subset in* C *and* ${\left\{z\mapsto b_{z}\right\}}_{z\in U}$ *a holomorphic family of fiberwise nondegenerate symmetric bilinear forms on E. For the bordism* $\left(M,\partial_{+}M,\partial_{-}M\right)$ *consider* ${\left\{{\left[\Delta ,\Omega_{B}\right]}_{\left(M,\partial_{+}M,\partial_{-}M\right)}^{E,g,b_{z}}\right\}}_{z\in O}$ *, the family of boundary value problems corresponding to bilinear Laplacians under absolute/relative boundary conditions, together with their* $L^{2}$ *-realizations denoted by* $\Delta_{B,b_{z}}$ *. Then, for each* $\psi \in \mathrm{End}(\Lambda T^{⁎}M⊗E)$ *, the map*

$$z\mapsto \underset{t\to 0}{\mathrm{LIM}}\left(S\mathrm{Tr}\left(\psi \exp(-t\Delta_{B,b_{z}})\right)\right)$$

*is holomorphic on O.*

Proof By compactness, we may assume without loss of generality that *ψ* is compactly supported in the interior of a sufficiently small open set *U* in *M*. Remark that the function $z\mapsto b_{z}^{-1}$ is holomorphic, since $z\mapsto b_{z}$ is a holomorphic family of fiberwise nondegenerate bilinear forms in z∈O. Then, as it can directly be checked by construction of the bilinear Laplacian in (2) and the boundary operators in (5), the assignments $z\mapsto \Delta_{E,g,b_{z}}$ and $z\mapsto B_{E,g,b_{z}}$ respectively define holomorphic functions in z∈O. Therefore, the coefficients of the symbols of $\Delta_{E,g,b_{z}}$ and $B_{E,g,b_{z}}$ are holomorphic functions in z∈O. Now, the expression ${\mathrm{LIM}}_{t\to 0}(S\mathrm{Tr}(\psi \exp(-t\Delta_{B,b_{z}})))$ is computed with the formula (25), by integrating the complex-valued function $S\mathrm{Tr}(\psi \cdot e_{m}(\Delta_{E,g,b_{z}}))$ over *U*, and the complex-valued function $S\mathrm{Tr}({\nabla_{\varsigma_{\mathrm{in}}}}^{k}\psi \cdot e_{m,k}(\Delta_{E,g,b_{z}},B_{E,g,b_{z}}))$ over U∩∂M. Since $e_{m}(\Delta_{E,g,b_{z}})$ are locally computable endomorphism invariants, the value of ${S\mathrm{Tr}}_{x}(\psi_{x}\cdot e_{m}{\left(\Delta_{E,g,b_{z}}\right)}_{x})$ can be computed inductively by using explicit formulas as a universal polynomial in terms of (finite number of the derivatives of) the coefficients of the symbol of $\Delta_{E,g,b_{z}}$, whenever these are given in local coordinates around at x∈M, see [24, Theorem 3], [23, formulas (3)–(6) and Lemma 1], see also [14, Section 2.6]. In the same way, since $e_{m,k}(\Delta_{E,g,b_{z}},B_{E,g,b_{z}})$ are locally computable endomorphism invariants on the boundary, the value of ${S\mathrm{Tr}}_{y}({\left({\nabla_{\varsigma_{\mathrm{in}}}}^{k}\psi \right)}_{y}\cdot e_{m,k}{\left(\Delta_{E,g,b_{z}},B_{E,g,b_{z}}\right)}_{y})$ is expressible, by inductively solving certain systems of ordinary differential equations, as a universal polynomial in terms of (finite number of the derivatives of) the coefficients of the symbols of $\Delta_{E,g,b_{z}}$ and $B_{E,g,b_{z}}$, whenever these are given in local coordinates around at y∈∂M, see [24, Theorem 3], [23, formulas (9)–(14) and Lemma 2], see also [14, Section 2.6]. Thus the mappings $z\mapsto {S\mathrm{Tr}}_{x}(e_{m}{\left(\Psi ,\Delta_{z}\right)}_{x})$ and $z\mapsto {S\mathrm{Tr}}_{x}(e_{m,k}{\left(\Psi ,\Delta_{z},B_{z}\right)}_{x})$ are holomorphic on *O* for each x∈U. Finally, by Moreraʼs Theorem, the integral of a function depending holomorphically on a parameter *z*, also depends holomorphically on *z*, that is, the function $z\mapsto {\mathrm{LIM}}_{t\to 0}(S\mathrm{Tr}(\psi \exp(-t\Delta_{B,b_{z}})))$ depends holomorphically on z∈O.  □

Theorem 2 *For* $\left(M,\partial_{+}M,\partial_{-}M\right)$ *consider the bilinear boundary value problem* ${\left[\Delta ,B\right]}_{\left(M,\partial_{+}M,\partial_{-}M\right)}^{E,g,b}$ *, together with its* $L^{2}$ *-realization* $\Delta_{B,b}$ *. If ϕ, ξ and Ψ are as in* Proposition 4 *, then*

(42) $$\underset{t\to 0}{\mathrm{LIM}}\left(S\mathrm{Tr}\left(\phi \exp(-t\Delta_{B,b})\right)\right)=\int_{M}\mathrm{Tr}(\phi )e(M,g)+{\left(-1\right)}^{m-1}\int_{\partial_{+}M}\mathrm{Tr}(\phi )i_{+}^{⁎}e_{b}(\partial M,g)-\int_{\partial_{-}M}\mathrm{Tr}(\phi )i_{-}^{⁎}e_{b}(\partial M,g),$$

*and*

(43) $$\underset{t\to 0}{\mathrm{LIM}}\left(S\mathrm{Tr}\left(-\Psi \exp(-t\Delta_{B,b})\right)\right)=-2\int_{M}{\frac{\partial }{\partial \tau }|}_{\tau =0}\tilde{e}(M,g,g+\tau g\xi )\wedge \omega \left(\nabla^{E},b\right)-2\int_{\partial_{+}M}{\frac{\partial }{\partial \tau }|}_{\tau =0}i_{+}^{⁎}{\tilde{e}}_{b}(\partial M,g,g+\tau g\xi )\wedge \omega \left(\nabla^{E},b\right)+\mathrm{rank}(E)\int_{\partial_{+}M}{\frac{\partial }{\partial \tau }|}_{\tau =0}i_{+}^{⁎}B(\partial M,g+\tau g\xi )-2{\left(-1\right)}^{m}\int_{\partial_{-}M}{\frac{\partial }{\partial \tau }|}_{\tau =0}i_{-}^{⁎}{\tilde{e}}_{b}(\partial M,g,g+\tau g\xi )\wedge \omega \left(\nabla^{E},b\right)+{\left(-1\right)}^{m+1}\mathrm{rank}(E)\int_{\partial_{-}M}{\frac{\partial }{\partial \tau }|}_{\tau =0}i_{-}^{⁎}B(\partial M,g+\tau g\xi ).$$

Proof By compactness of *M*, it suffices to show that each point x∈M admits a neighborhood *U* so that the formulas above hold for all *ϕ* with supp(ϕ)⊂U and *ξ* with supp(ξ)⊂U. For each x∈M, choose $b_{z}=b+z\tilde{b}$, $h_{s}$ and *U* as in Proposition 6, with supp(ϕ)⊂U. By Proposition 6(iii), we obtain ${\mathrm{LIM}}_{t\to 0}\;S\mathrm{Tr}(\phi \exp(-t\Delta_{B,b_{s-i}}))={\mathrm{LIM}}_{t\to 0}\;S\mathrm{Tr}(\phi \exp(-t\Delta_{B,h_{s}}))$, for all |s|≤1, for these quantities depend on the geometry over *U* only. From Theorem 1, we have

$$\underset{t\to 0}{\mathrm{LIM}}S\mathrm{Tr}\left(\phi \exp(-t\Delta_{B,b_{s-i}})\right)=\int_{M}\mathrm{Tr}(\phi )e(M,g)+{\left(-1\right)}^{m-1}\int_{\partial_{+}M}\mathrm{Tr}(\phi )i_{+}^{⁎}e_{b}(\partial M,g)-\int_{\partial_{-}M}\mathrm{Tr}(\phi )i_{-}^{⁎}e_{b}(\partial M,g)$$

for all |s|≤1. Now, since the function $z\mapsto {\mathrm{LIM}}_{t\to 0}\;S\mathrm{Tr}(\phi \exp(-t\Delta_{B,b_{z}}))$ depends holomorphically on *z* (see Lemma 10), that the right hand side of the equality above is constant in *z*, and that the domain of definition of *z* contains an accumulation point, these formulas are extended by analytic continuation to

$$\underset{t\to 0}{\mathrm{LIM}}S\mathrm{Tr}\left(\phi \exp(-t\Delta_{B,b_{z}})\right)=\int_{M}\mathrm{Tr}(\phi )e(M,g)+{\left(-1\right)}^{m-1}\int_{\partial_{+}M}\mathrm{Tr}(\phi )i_{+}^{⁎}e_{b}(\partial M,g)-\int_{\partial_{-}M}\mathrm{Tr}(\phi )i_{-}^{⁎}e_{b}(\partial M,g),$$

for all $|z|\leq \sqrt{2}$. After setting z=0 we obtain the desired identity in (42). We now show (43). Similarly take *ξ* with supp(ξ)⊂U, using Proposition 6(iii), we obtain

(44) $$\underset{t\to 0}{\mathrm{LIM}}S\mathrm{Tr}\left(-\Psi \exp(-t\Delta_{B,b_{s-i}})\right)=\underset{t\to 0}{\mathrm{LIM}}S\mathrm{Tr}\left(-\Psi \exp(-t\Delta_{B,h_{s}})\right)$$

for all |s|≤1, for these quantities depend on the geometry over *U* only. Then, we apply Theorem 1 to the right hand side of the equality in (44) we conclude

(45) $$\underset{t\to 0}{\mathrm{LIM}}S\mathrm{Tr}\left(-\Psi \exp(-t\Delta_{B,b_{s-i}})\right)=-2\int_{M}{\frac{\partial }{\partial \tau }|}_{\tau =0}\tilde{e}(M,g,g+\tau g\xi )\wedge \omega \left(\nabla^{E},b_{s-i}\right)-2\int_{\partial_{+}M}{\frac{\partial }{\partial \tau }|}_{\tau =0}i_{+}^{⁎}{\tilde{e}}_{b}(\partial M,g,g+\tau g\xi )\wedge \omega \left(\nabla^{E},b_{s-i}\right)+\mathrm{rank}(E)\int_{\partial_{+}M}{\frac{\partial }{\partial \tau }|}_{\tau =0}i_{+}^{⁎}B(\partial M,g+\tau g\xi )-2{\left(-1\right)}^{m}\int_{\partial_{-}M}{\frac{\partial }{\partial \tau }|}_{\tau =0}i_{-}^{⁎}{\tilde{e}}_{b}(\partial M,g,g+\tau g\xi )\wedge \omega \left(\nabla^{E},b_{s-i}\right)+{\left(-1\right)}^{m+1}\mathrm{rank}(E)\int_{\partial_{-}M}{\frac{\partial }{\partial \tau }|}_{\tau =0}i_{-}^{⁎}B(\partial M,g+\tau g\xi ),$$

for all |s|≤1. Now, the function $z\mapsto {\mathrm{LIM}}_{t\to 0}\;S\mathrm{Tr}(\phi \exp(-t\Delta_{B,b_{z}}))$ on the left of (45) depends holomorphically on *z* see Lemma 10. On the other hand the long expression on the right hand side of the equality above in (45) is also a holomorphic function in z∈C with $|z|\leq \sqrt{2}$, since it can be formally considered as the composition of constant functions (in *z*) and the function $z\mapsto \omega (\nabla^{E},b_{z})=-\frac{1}{2}\mathrm{Tr}(b_{z}^{-1}\nabla^{E}b_{z})$, which is holomorphic, since by Proposition 6 the bilinear form $b_{z}$ in (38) is fiberwise nondegenerate for $|z|\leq \sqrt{2}$. Then the identity in (45) can be analytically extended to

(46) $$\underset{t\to 0}{\mathrm{LIM}}S\mathrm{Tr}\left(-\Psi \exp(-t\Delta_{B,b_{z-i}})\right)=-2\int_{M}{\frac{\partial }{\partial \tau }|}_{\tau =0}\tilde{e}(M,g,g+\tau g\xi )\wedge \omega \left(\nabla^{E},b_{z-i}\right)-2\int_{\partial_{+}M}{\frac{\partial }{\partial \tau }|}_{\tau =0}i_{+}^{⁎}{\tilde{e}}_{b}(\partial M,g,g+\tau g\xi )\wedge \omega \left(\nabla^{E},b_{z-i}\right)+\mathrm{rank}(E)\int_{\partial_{+}M}{\frac{\partial }{\partial \tau }|}_{\tau =0}i_{+}^{⁎}B(\partial M,g+\tau g\xi )-2{\left(-1\right)}^{m}\int_{\partial_{-}M}{\frac{\partial }{\partial \tau }|}_{\tau =0}i_{-}^{⁎}{\tilde{e}}_{b}(\partial M,g,g+\tau g\xi )\wedge \omega \left(\nabla^{E},b_{z-i}\right)+{\left(-1\right)}^{m+1}\mathrm{rank}(E)\int_{\partial_{-}M}{\frac{\partial }{\partial \tau }|}_{\tau =0}i_{-}^{⁎}B(\partial M,g+\tau g\xi ),$$

for z∈C with $|z-i|\leq \sqrt{2}$. Finally (43) follows from setting z=i into (46) and then $b_{0}=b$ follows from (38).  □

## 3 Complex-valued analytic torsion on compact bordisms

Let $\left(M,\partial_{+}M,\partial_{-}M\right)$ be a Riemannian bordism and *E* be complex flat vector bundle over *M* endowed with a nondegenerate symmetric bilinear form. Consider $\Delta_{B}$ the $L^{2}$-realization of the bilinear Laplacian acting on *E*-valued smooth forms satisfying absolute boundary conditions on $\partial_{+}M$ and relative ones on $\partial_{-}M$.

If $\Omega_{\Delta_{B}}(0)$ is the 0-generalized eigenspace of $\Delta_{B}$, consider the restriction of $\beta_{g,b}$ to $\Omega_{\Delta_{B}}(0)$; this is a nondegenerate symmetric bilinear form in view of Proposition 1. By [4, Lemma 3.3] we obtain a nondegenerate bilinear form on $\det\;H(\Omega_{\Delta_{B}}(0))$, which in turn, by Proposition 3, induces a bilinear form on $\det(H(M,\partial_{-}M;E))$, which we denote by $\tau {\left(0\right)}_{E,g,b}$. Let us denote by

$$\Delta_{B,q}^{c}:=\Delta_{B}{|}_{\Omega_{\Delta_{B}}^{q}(M;E){\left(0\right)}^{c}{|}_{B}}$$

the restriction of $\Delta_{B}$ to $\Omega_{\Delta_{B}}^{q}(M;E){\left(0\right)}^{c}{|}_{B}$, i.e., the space of smooth differential forms of degree *q* which are not in $\Omega_{\Delta_{B}}(M;E)(0)$ but satisfy boundary conditions. Lemma 2 permits us to choose a non-zero Agmon angle avoiding the spectrum of $\Delta_{B,q}^{c}$ so that complex powers of the bilinear Laplacian can be defined. Then, the function $s\mapsto {\left(\Delta_{B,q}^{c}\right)}^{-s}$ associates to each s∈C, with Re(s)>dim(M)/2, an operator of Trace class and it extends to a meromorphic function on the complex plane which is holomorphic at 0, see [14], [22], [23], [24] or more generally, for pseudo-differential boundary value problems, see [15, Chapter 4]. The *ζ*-regularized determinant of $\Delta_{B,q}$ is defined as

$$\det^{\prime }(\Delta_{B,q}):=\exp\left(-{\frac{\partial }{\partial s}|}_{s=0}\mathrm{Tr}\left({\left(\Delta_{B,q}^{c}\right)}^{-s}\right)\right).$$

From Lemma 2 this determinant does not depend on the choice of the Agmonʼs angle. By using [4, Lemma 3.3], the complex-valued Ray–Singer torsion on the bordism $\left(M,\partial_{+}M,\partial_{-}M\right)$ is defined as the bilinear form on the determinant line $\det\;H(M,\partial_{-}M;E)$ given by

$$\tau_{E,g,b}:=\tau {\left(0\right)}_{E,g,b}\prod_{q}{\left(\det^{\prime }(\Delta_{B,q})\right)}^{{\left(-1\right)}^{q}q}.$$

The following generalizes the formulas obtained in [4] in the case without boundary and they are based on the corresponding ones for the Ray–Singer metric in [8]. They also coincide with the ones obtained by Su in odd dimensions, but they do not require that the smooth variations of *g* and *b* are supported on a compactly supported in the interior of *M*, see [25].

Theorem 3 **Anomaly formulas**

*Let*$\left(M,\partial_{+}M,\partial_{-}M\right)$*be a compact Riemannian bordism and E be complex flat vector bundle over M. Consider*$g_{u}$*a smooth one-parameter family of Riemannian metrics on M and*$b_{u}$*a smooth one-parameter family of a fiberwise nondegenerate symmetric bilinear forms on E and denote by*${\dot{g}}_{t}$*and*${\dot{b}}_{t}$*their corresponding infinitesimal variations. Let*$\tau_{E,g_{u},b_{u}}$*the associated family of complex valued analytic torsions. Then, we have the following logarithmic derivative*

$${\frac{\partial }{\partial w}|}_{u}{\left(\frac{\tau_{E,g_{w},b_{w}}}{\tau_{E,g_{u},b_{u}}}\right)}^{2}=E(b_{u},g_{u})+\tilde{E}(b_{u},g_{u})+B(g_{u}),$$

*where*$\omega (\nabla^{E},b):=-\frac{1}{2}\mathrm{Tr}(b^{-1}\nabla^{E}b)$*is the Kamber–Tondeur form, see*[4, *Section* 2.4]*and*

$$E(b_{u},g_{u}):=\int_{M}\mathrm{Tr}\left(b_{u}^{-1}{\dot{b}}_{u}\right)e(M,g)+{\left(-1\right)}^{m-1}\int_{\partial_{+}M}\mathrm{Tr}\left(b_{u}^{-1}{\dot{b}}_{u}\right)e_{b}(\partial M,g_{u})-\int_{\partial_{-}M}\mathrm{Tr}\left({b^{\prime }}_{u}^{-1}{\dot{b}}_{u}^{\prime }\right)e_{b}(\partial M,g_{u}),$$

$$\tilde{E}(b_{u},g_{u}):=-2\int_{M}{\frac{\partial }{\partial t}|}_{t=0}\tilde{e}(M,g_{u},g_{u}+t{\dot{g}}_{u})\wedge \omega \left(\nabla^{E},b_{u}\right)-2\int_{\partial_{+}M}{\frac{\partial }{\partial t}|}_{t=0}i_{+}^{⁎}{\tilde{e}}_{b}(\partial M,g_{u},g_{u}+t{\dot{g}}_{u})\wedge \omega \left(\nabla^{E},b_{u}\right)-2{\left(-1\right)}^{m}\int_{\partial_{-}M}{\frac{\partial }{\partial t}|}_{t=0}i_{-}^{⁎}{\tilde{e}}_{b}(\partial M,g_{u},g_{u}+t{\dot{g}}_{u})\wedge \omega \left(\nabla^{E},b_{u}\right),$$

$$B(g_{u}):=\mathrm{rank}(E)\int_{\partial_{+}M}{\frac{\partial }{\partial t}|}_{t=0}i_{+}^{⁎}B(\partial M,g_{u}+t{\dot{g}}_{u})+{\left(-1\right)}^{m+1}\mathrm{rank}(E)\int_{\partial_{-}M}{\frac{\partial }{\partial t}|}_{t=0}i_{-}^{⁎}B(\partial M,g_{u}+t{\dot{g}}_{u}),$$

Proof The method described in [4, Section 6] leading to the infinitesimal variation of the torsion in the closed situation also holds in the situation with boundary; this was also used in [25]. In particular, by [4, formula (54)], the problem of computing this infinitesimal variation boils down to computing ${\mathrm{LIM}}_{t\to 0}(S\mathrm{Tr}(\phi \exp(-t\Delta_{B})))$ and ${\mathrm{LIM}}_{t\to 0}(S\mathrm{Tr}(-\Psi \exp(-t\Delta_{B})))$ associated to $\Delta_{B}$ with $\phi =b_{u}^{-1}{\dot{b}}_{u}$ and $\xi =g_{u}^{-1}{\dot{g}}_{u}$ respectively given by (42), (43) in Theorem 2.  □

## Appendix A

In this appendix, for the readerʼs convenience, we stay close to the notation in [8] (see also [7, Chapter 3]) and we briefly recall the definition of the characteristic forms appearing in the anomaly formulas in Sections 2, 3.

### A.1 The Berezin integral and Pfaffian

For *A* and *B* two unital $Z_{2}$-graded algebras, with respective unities $1_{A}$ and $1_{B}$, we consider their $Z_{2}$-*graded tensor product* denoted by $A\hat{⊗}B$. The map $w\mapsto w\hat{⊗}1_{B}$ provides a canonical isomorphism between *A* and the subalgebra $A\hat{⊗}1_{B}\subset A\hat{⊗}B$, whereas with the map $w\mapsto \hat{w}:=1_{A}\hat{⊗}w$ we canonically identify *B* with the subalgebra $\hat{B}:=1_{A}\hat{⊗}B\subset A\hat{⊗}B$. As $Z_{2}$-graded algebras, one has $A\hat{⊗}\hat{B}≅A\hat{⊗}B$.

Let *W* and *V* be finite dimensional vector spaces of dimension *n* and *l* respectively, with $W^{\prime }$ and $V^{\prime }$ their corresponding dual spaces. We denote by $\Theta_{W}$ the orientation line of *W*. Assume *W* is endowed with a Hermitian product 〈⋅,⋅〉, fix ${\left\{w_{i}\right\}}_{i=1}^{n}$ an orthonormal basis of *W* and use the metric to fix ${\left\{w^{i}\right\}}_{i=1}^{n}$ the corresponding dual basis in $W^{\prime }$. Then, each antisymmetric endomorphism *K* of *W* can be uniquely identified with the section **K** of $\hat{\Lambda (W^{\prime })}$ given by $K:=\frac{1}{2}\sum_{1\leq i,j\leq n}\langle w_{i},Kw_{j}\rangle \hat{w^{i}}\wedge \hat{w^{j}}$.

The *Berezin integral* $\int^{B}:\Lambda V^{\prime }\hat{⊗}\hat{\Lambda (W^{\prime })}\to \Lambda V^{\prime }⊗\Theta_{W}$ is the linear map given by $\alpha \hat{⊗}\hat{\beta }\mapsto C_{B}\beta_{g,b}(w_{1},\ldots ,w_{n})$, with constant $C_{B}:={\left(-1\right)}^{n(n+1)/2}\pi^{-n/2}$. Then, Pf(K/2π), the *Pfaffian* of K/2π, is defined by

$$\mathrm{Pf}(K/2\pi ):=\int^{B}\exp(K/2\pi ).$$

Remark that Pf(K/2π)=0, if *n* is odd. By standard fiberwise considerations the map **Pf** is extended for vector bundles over *M*.

### A.2 Certain characteristic forms on the boundary

Let *M* be an *m*-dimensional compact Riemannian manifold with boundary ∂*M* and denote by i:∂M↪M the canonical embedding. We denote by $g:=g^{\mathrm{TM}}$ (resp. $g^{\partial }:=g^{T\partial M}$) the Riemannian metric on *TM* (resp. on T∂M and induced by *g*), by ∇ (resp. $\nabla^{\partial }$) the corresponding Levi-Cività connection and by $R^{\mathrm{TM}}$ (resp. $R^{T\partial M}$) its curvature. Let ${\left\{e_{i}\right\}}_{i=1}^{m}$ be an orthonormal frame of *TM* with the property that near the boundary, $e_{m}=\varsigma_{\mathrm{in}}$, i.e., the inwards pointing geodesic unit normal vector field on the boundary. The corresponding induced orthonormal local frame on T∂M will be denoted by ${\left\{e_{\alpha }\right\}}_{\alpha =1}^{m-1}$. As usual, the metric is used to fix ${\left\{e^{i}\right\}}_{i=1}^{m}$ (resp. ${\left\{e^{\alpha }\right\}}_{\alpha =1}^{m-1}$) the corresponding dual frame of $T^{⁎}M$ (resp. $T^{⁎}\partial M$).

With the notation in Appendix A.1, a smooth section *w* of $\Lambda T^{⁎}M$ is identified with the section $w\hat{⊗}1$ of $\Lambda T^{⁎}M\hat{⊗}\Lambda T^{⁎}M$, whereas $\hat{w}$ denotes the corresponding section $1\hat{⊗}w$ of $\Lambda T^{⁎}M\hat{⊗}\Lambda T^{⁎}M$.

Here, the Berezin integrals $\int^{B_{M}}:\Lambda T^{⁎}M\hat{⊗}\hat{\Lambda T^{⁎}M}\to \Lambda T^{⁎}M⊗\Theta_{M}$ and $\int^{B_{\partial M}}:\Lambda T^{⁎}\partial M\hat{⊗}\hat{\Lambda (T^{⁎}\partial M)}\to \Lambda T^{⁎}\partial M⊗\Theta_{\partial M}$ can be compared under the given convention for the induced orientation bundle on the boundary, see Section 1.

The curvature $R^{\mathrm{TM}}$ associated to ∇, considered as a smooth section of $\Lambda^{2}(T^{⁎}M)\hat{⊗}\hat{\Lambda^{2}(T^{⁎}M})\to M$, can be expanded in terms of the frame above as

$$R^{\mathrm{TM}}:=\frac{1}{2}\sum_{1\leq k,l\leq m}g^{\mathrm{TM}}\left(e_{k},R^{\mathrm{TM}}e_{l}\right)\hat{e^{k}}\wedge \hat{e^{l}}\in \Gamma \left(M;\Lambda^{2}\left(T^{⁎}M\right)\hat{⊗}\hat{\Lambda^{2}\left(T^{⁎}M\right)}\right).$$

In the same way, consider the forms (47)

$$i^{⁎}R^{\mathrm{TM}}:=\frac{1}{2}\sum_{1\leq k,l\leq m}g^{\mathrm{TM}}\left(e_{k},i^{⁎}R^{\mathrm{TM}}e_{l}\right)\hat{e^{k}}\wedge \hat{e^{l}}\in \Gamma \left(\partial M;\Lambda^{2}\left(T^{⁎}\partial M\right)\hat{⊗}\hat{\Lambda^{2}\left(T^{⁎}M\right)}\right),$$

$${R^{\mathrm{TM}}|}_{\partial M}:=\frac{1}{2}\sum_{1\leq \alpha ,\beta \leq m-1}g^{\mathrm{TM}}\left(e_{\alpha },i^{⁎}R^{\mathrm{TM}}e_{\beta }\right)\hat{e^{\alpha }}\wedge \hat{e^{\beta }}\in \Gamma \left(\partial M;\Lambda^{2}\left(T^{⁎}\partial M\right)\hat{⊗}\hat{\Lambda^{2}\left(T^{⁎}(\partial M)\right)}\right),$$

$$R^{T\partial M}:=\frac{1}{2}\sum_{1\leq \alpha ,\beta \leq m-1}g^{T\partial M}\left(e_{\alpha },R^{T\partial M}e_{\beta }\right)\hat{e^{\alpha }}\wedge \hat{e^{\beta }}\in \Gamma \left(\partial M;\Lambda^{2}\left(T^{⁎}\partial M\right)\hat{⊗}\hat{\Lambda^{2}\left(T^{⁎}(\partial M)\right)}\right),$$

$$S:=\frac{1}{2}\sum_{\beta =1}^{m-1}g^{\mathrm{TM}}\left(\left(i^{⁎}\nabla^{\mathrm{TM}}\right)\varsigma_{\mathrm{in}},e_{\beta }\right)\hat{e^{\beta }}\in \Gamma \left(\partial M;T^{⁎}\partial M\hat{⊗}\hat{\Lambda^{1}\left(T^{⁎}(\partial M)\right)}\right)$$

 to define (48)

$$e\left(M,\nabla^{TM}\right):=\int^{B_{M}}\exp\left(-\frac{1}{2}R^{TM}\right),$$

$$e\left(\partial M,\nabla^{T\partial M}\right):=\int^{B_{\partial M}}\exp\left(-\frac{1}{2}R^{T\partial M}\right),$$

$$e_{b}\left(\partial M,\nabla^{TM}\right):={\left(-1\right)}^{m-1}\int^{B_{\partial M}}\exp\left(-\frac{1}{2}\left({R^{TM}|}_{\partial M}\right)\right)\sum_{k=0}^{\infty }\frac{S^{k}}{2\Gamma (\frac{k}{2}+1)},$$

$$B\left(\partial M,\nabla^{TM}\right):=-\int_{0}^{1}\frac{du}{u}\int^{B_{\partial M}}\exp\left(-\frac{1}{2}R^{T\partial M}-u^{2}S^{2}\right)\sum_{k=1}^{\infty }\frac{{\left(uS\right)}^{k}}{2\Gamma (\frac{k}{2}+1)}.$$

### A.3 Secondary characteristic forms

Given ${\left\{g_{s}:=g_{s}^{\mathrm{TM}}\right\}}_{s\in R}$ (resp. ${\left\{g_{s}^{\partial }:=g_{s}^{T\partial M}\right\}}_{s\in R}$) a smooth family of Riemannian metrics on *TM* (resp. the induced family of metrics on T∂M), we sketch the construction in [8] (see also [7, (4.53)]) for the (secondary) *Chern–Simons forms* $\tilde{e}(M,g_{0},g_{s})$ and ${\tilde{e}}_{b}(\partial M,g_{0},g_{s})$.

Let $\nabla_{s}:=\nabla_{g_{s}}^{\mathrm{TM}}$ and $R_{s}:=R_{g_{s}}^{\mathrm{TM}}$ (resp. $\nabla_{s}^{\partial }:=\nabla_{g_{s}^{\partial }}^{T\partial M}$ and $R_{s}^{\partial }:=R_{g_{s}^{\partial }}^{T\partial M}$) be the Levi-Cività connections and curvatures on *TM* (resp. on T∂M) associated to the metrics $g_{s}$ (resp. $g_{s}^{\partial }$). Consider the *deformation spaces* $\tilde{M}:=M\times R$ (resp. $\tilde{\partial M}:=\partial M\times R$) with $\pi_{\tilde{M}}:\tilde{M}\to R\text{ and }p_{M}:\tilde{M}\to M$, its canonical projections (resp. $\pi_{\tilde{\partial M}}:\tilde{\partial M}\to R\text{ and }p_{\partial M}:\tilde{\partial M}\to \partial M$). If $\tilde{i}:=i\times id_{R}:\tilde{\partial M}\to \tilde{M}$ is the natural embedding induced by i:∂M→M, then $\pi_{\tilde{\partial M}}=\pi_{\tilde{M}}\circ \tilde{i}$. The *vertical bundle* of the fibration $\pi_{\tilde{M}}:\tilde{M}\to R$ (resp. $\pi_{\tilde{\partial M}}:\tilde{\partial M}\to R$) is the pull-back of the tangent bundle TM→M along $p_{M}:\tilde{M}\to M$ (resp. the pull-back of T∂M→∂M along $p_{\partial M}:\tilde{\partial M}\to \partial M$), i.e.,

(49) $$\mathrm{TM}:=p_{M}^{⁎}TM\to \tilde{M}\;\left(\text{resp. }T\partial M:=p_{\partial M}^{⁎}T\partial M\to \tilde{\partial M}\right)$$

and it is considered as a subbundle of $T\tilde{M}$ (resp. $T\tilde{\partial M}$). The bundle TM (resp. T∂M) in (49) is naturally equipped with a Riemannian metric $g^{\mathrm{TM}}$ which coincides with $g_{s}$ (resp. $g_{s}^{\partial }$) at M×{s} (resp. ∂M×{s}), for which there exists a unique natural metric connection $\nabla^{\mathrm{TM}}$ (resp. $\nabla^{T\partial M}$) whose curvature tensor is denoted by $R^{\mathrm{TM}}$ (resp. $R^{T\partial M}$); for more details, see [8, Section 1.5, (1.44) and Definition 1.1], and also [7, (4.50) and (4.51)]. Near the boundary, consider orthonormal frames of TM such that $e_{m}(y,s)=\varsigma_{\mathrm{in}}$ for each y∈∂M with respect to the metric $g_{s}$. Finally, by using the formalism described above associated to $R^{\mathrm{TM}}$ and $R^{T\partial M}$ to define (48), if ${\mathrm{incl}}_{s}:M\to \tilde{M}$ is the inclusion map given by ${\mathrm{incl}}_{s}(x)=(x,s)$ for $x_{0}\in M$ and s∈R, then one defines (50)

$$\tilde{e}(M,g_{0},g_{\tau }):=\int_{0}^{\tau }{\mathrm{incl}}_{s}^{⁎}\left(\iota \left(\frac{\partial }{\partial s}\right)e\left(\tilde{M},\nabla^{\mathrm{TM}}\right)\right)\;ds\in \Omega^{m-1}(M,\Theta_{M}),$$

$${\tilde{e}}_{b}(\partial M,g_{0},g_{\tau }):=\int_{0}^{\tau }{\mathrm{incl}}_{s}^{⁎}\left(\iota \left(\frac{\partial }{\partial s}\right)e_{b}\left(\tilde{\partial M},\nabla^{\mathrm{TM}}\right)\right)\;ds\in \Omega^{m-2}(\partial M,\Theta_{M}),$$

 where ι(X) indicates the contraction with respect to the vector field *X*.
